# Targeting oxidative phosphorylation with lixumistat overcomes PARP inhibitor resistance in ovarian cancer

**DOI:** 10.1038/s41419-026-09140-y

**Published:** 2026-08-07

**Authors:** Sa Deok Hong, Nar Bahadur Katuwal, Mithun Ghosh, Seong Min Park, Ju Hyun Kim, Seul-Gi Kim, Min Sil Kang, Yong Wha Moon

**Affiliations:** 1 Department of Biomedical Science, The Graduate School, CHA University, Seongnam-si, Republic of Korea; 2 Hematology and Oncology, CHA Bundang Medical Center, CHA University, Seongnam-si, Republic of Korea

**Keywords:** Ovarian cancer, Cancer therapeutic resistance, Targeted therapies

## Abstract

Ovarian cancer remains the leading cause of gynecologic cancer mortality, yet nearly half of BRCA1/2-mutated ovarian cancer patients eventually develop resistance to poly(ADP-ribose) polymerase (PARP) inhibitors. In this study, we investigated whether lixumistat, a novel oxidative phosphorylation (OXPHOS) inhibitor, could overcome PARP inhibitor resistance in preclinical BRCA1/2-mutated ovarian cancer models. Long-term olaparib exposure generated olaparib-resistant BRCA-mutated ovarian cancer cell lines and patient-derived tumor xenograft (PDTX) models. Olaparib-resistant cells exhibited elevated OXPHOS activity. Lixumistat, when combined with olaparib, demonstrated synergistic antitumor effects in both olaparib-sensitive and -resistant cell lines and PDTX models. Switching from olaparib monotherapy to combination markedly suppressed tumor growth in partially resistant and acquired-resistant PDTX models. These results suggest that early integration of lixumistat could enhance efficacy and delay PARP inhibitor resistance. Mechanistically, lixumistat restores PARP inhibitor sensitivity by inhibiting the OXPHOS–NAD⁺-poly(ADP)-ribosylation (PARylation) axis, which reduces NAD⁺ availability and subsequent PARylation. Exogenous NAD⁺ reversed these effects. Additionally, lixumistat downregulated the SNAIL-mediated IL6-phosphorylated STAT3 and phosphorylated retinoblastoma pathway, impairing cell-cycle progression and further enhancing PARP inhibitor efficacy. Together, these findings identify a novel OXPHOS-associated mechanism underlying PARP inhibitor resistance and establish lixumistat as a promising combinatorial strategy to potentiate PARP inhibitors in both PARP inhibitor-naive and -resistant ovarian cancer.

## Introduction

Ovarian cancer is the eighth most common cancer and the leading cause of gynecologic cancer death worldwide. Despite therapeutic advances, the 5-year survival rate remains below 50% due to its aggressive behavior and high recurrence rate [1]. Standard treatment involves cytoreductive surgery followed by platinum- and taxane-based chemotherapy, but most patients relapse within two years, and responses to subsequent treatments are limited [2]. To improve long-term outcomes, maintenance therapies have been introduced after first-line chemotherapy. Bevacizumab, added to and maintained after chemotherapy, modestly improved progression-free survival in high-risk patients in the GOG-0218 [3] and ICON7 trials [4]. Following chemotherapy, poly(ADP-ribose) polymerase (PARP) inhibitor maintenance has shown greater benefit; the SOLO1 trial [5] reported significantly prolonged progression-free survival with olaparib in breast cancer gene (BRCA)1/2-mutated patients and the PRIMA trial [6] demonstrated niraparib efficacy in high-risk patients regardless of BRCA status [7]. However, most patients eventually experience a relapse due to acquired resistance to PARP inhibitors. Therefore, new therapeutic strategies need to be developed.

PARP1 plays a key role in base excision repair, a DNA repair pathway for resolving single-strand breaks [8]. In BRCA1/2-mutated tumors, which are homologous recombination (HR) deficient, PARP inhibition causes impaired DNA damage and cell death through synthetic lethality. Up to 18% of epithelial ovarian cancers have germline BRCA1/2 mutations [9], making them sensitive to PARP inhibitors. Despite the clinical success of PARP inhibitors in HR-deficient ovarian cancer, PARP inhibitors encounter major challenges due to resistance. Around 40–50% of BRCA1/2-mutated patients show intrinsic or acquired resistance [10], and 40–70% of initial responders develop acquired resistance [8, 11]. Underlying mechanisms for PARP inhibitor resistance include DNA damage repair through HR restoration, BRCA reversion mutations, increased NAD⁺ metabolism, PARG loss, poly(ADP)-ribosylation (PARylation) restoration, PARP1 mutations and post-translational modifications such as PRMT1-mediated PARP1 methylation [8, 12–14]. These mechanisms highlight the need for mechanistic understanding of PARP inhibitor resistance and combination strategies to overcome resistance.

Since Otto Warburg first identified that cancer cells preferentially produce ATP via aerobic glycolysis—a phenomenon termed the Warburg effect [15]—cancer metabolism has become an area of intense scientific interest. Although glycolysis is a dominant metabolic pathway in cancer, recent studies have shown that mitochondrial oxidative phosphorylation (OXPHOS) is also upregulated in various cancers, including ovarian, endometrial, colorectal, prostate, head and neck, thyroid, and hematological malignancies [16]. OXPHOS generates ATP through the electron transport chain in the mitochondrial membrane, and its activation supports not only energy production but also redox homeostasis and biosynthesis [17]. Notably, elevated OXPHOS activity has been linked to poor prognosis and resistance to chemotherapies [18]. This suggests that OXPHOS activation may act as a survival mechanism under therapeutic stress. Several OXPHOS inhibitors, such as biguanides and other mitochondrial complex-targeting agents, are being evaluated in preclinical and clinical studies [16, 19]. The emerging correlation between OXPHOS activity and drug resistance suggests that mitochondrial metabolism inhibition potentiates the overcoming of drug resistance, including PARP inhibitor resistance [13]. A novel biguanide derivative compound, lixumistat, is a selective OXPHOS inhibitor that targets mitochondrial complex I [20]. Previous studies have shown that lixumistat inhibits cancer progression [20, 21] and biguanides enhance the efficacy of therapies, including PARP inhibitors [22, 23], with good in vivo tolerability. These findings indicate that lixumistat has the potential to serve as a combination strategy to overcome PARP inhibitor resistance.

As mentioned above, PARP inhibitor resistance is still a major challenge in the treatment of ovarian cancer. This study aimed to investigate whether OXPHOS inhibition by lixumistat enhances sensitivity to PARP inhibitors. To explore this, we utilized the olaparib-resistant ovarian cancer cells and patient-derived tumor xenograft (PDTX) models and evaluated the effects of lixumistat using in vitro and in vivo assays. Our findings demonstrate that lixumistat effectively overcomes PARP inhibitor resistance and highlight its potential as a promising combinatorial therapeutic strategy.

## Materials and methods

### Drugs

Olaparib (cat# CS-0075) and talazoparib (cat# CS-0937) were purchased from ChemScene (Monmouth, NJ, USA) and dissolved in dimethyl sulfoxide (Sigma-Aldrich, St. Louis, MO, USA). Lixumistat (IM156) was provided by Immunomet Therapeutics, Inc. (Houston, TX, USA) for non-commercial research purposes, and it was initially dissolved in distilled water.

### Cell lines

SNU-251 (BRCA1-mutated, RRID: CVCL_5040) and PEO1 (BRCA2-mutated, RRID: CVCL_2686) were purchased from the Korean Cell Line Bank (Seoul, Korea) and ECACC (Salisbury, UK), respectively. HEK293T (RRID: CVCL_0063) and BEAS-2B (RRID: CVCL_0168) were used as non-malignant control cell lines. SNU-251 cells were cultured in RPMI 1640 medium (Welgene, Daegu, Korea), while HEK293T and BEAS-2B cells were maintained in DMEM (Welgene). All media were supplemented with 10% heat-inactivated FBS and 1% penicillin/streptomycin. PEO1 cells were additionally supplemented with 2 mM sodium pyruvate (Thermo Fisher Scientific, Waltham, MA, USA). All cells were maintained at 37°C in a humidified atmosphere containing 5% CO_2_.

### Olaparib-resistant cell line establishment

Olaparib-resistant cell lines were established by gradually increasing the olaparib concentration as previously described [24], starting from 1/100 of half-maximal inhibitory concentration (IC_50_), which was 3.7 µM for SNU-251 and 20 µM for PEO1 cells, respectively. The culture medium containing the drug was refreshed every 3–4 days. Once the cells were able to proliferate stably at a given concentration, the olaparib dose was increased stepwise. After approximately 6–9 months, olaparib-resistant derivatives were established, exhibiting a 12–13-fold increase in the IC_50_ compared to parental cells. These resistant cell lines were designated SNU251-OR and PEO1-OR, representing olaparib-resistant SNU-251 and PEO1 cells, respectively. Before performing any experiment, the drug was washed out for at least 48 h (hour) and maintained in a fresh drug-free medium.

### Cell viability assay

Cell viability was assessed using the thiazolyl blue tetrazolium bromide (MTT; Sigma, St. Louis, MO, USA) colorimetric assay. Cells in the logarithmic phase were seeded in 96-well plates at a density of 1000–3000 cells per well. After allowing the cells to adhere to the dish for 24 h, various concentrations of olaparib or lixumistat were added to the cells, followed by incubation at 37 °C for 72 h. Subsequently, MTT solution (5 mg/mL) was added to each well and incubated for 4 h. Medium was then removed. 100 µL of dimethyl sulfoxide was added to dissolve the formazan crystals. Finally, the optical density was measured at 540 nm using a microplate reader (Multiskan FC, Thermo Fisher Scientific, Waltham, MA, USA). CompuSyn software (ComboSyn, Inc., NJ, USA, RRID:SCR_022931) was used to calculate IC_50_ values.

### Western blot analysis

Cells were collected and rinsed with phosphate-buffered saline (PBS; Welgene), then lysed at 4 °C for 1 h using radioimmunoprecipitation assay buffer (cat# 89901, Thermo Fisher Scientific) supplemented with a protease inhibitor cocktail (cat# 11873580001, Roche, Basel, Switzerland) and phosphatase inhibitors (cat# 1862495, Thermo Fisher Scientific). The lysates were separated by SDS-PAGE and transferred onto polyvinylidene difluoride membranes (cat# IPVH00010, Millipore, Burlington, MA, USA). Membranes were blocked at room temperature for 1 h using 5% skim milk in Tris-buffered saline containing 0.1% Tween 20. Primary antibodies were incubated overnight at 4 °C, followed by incubation with Horseradish peroxidase-conjugated secondary antibodies at room temperature for 1 h. Protein bands were visualized using Detection Reagent 1 (peroxide solution, cat# 1859700) and Detection Reagent 2 (luminol enhancer, cat# 1859697), both from Thermo Fisher Scientific. Image acquisition was performed using GeneSys software (RRID: SCR_015770).

A complete list of antibodies used in this study is provided in Supplementary Table 1.

### Apoptosis assay

Apoptosis was assessed by flow cytometry using Annexin V/propidium iodide (PI) staining. Following trypsinization, cells were washed twice with PBS and pelleted by centrifugation at 500 × *g*. The cells were then resuspended at a concentration of 1 × 10⁶ cells/mL in Annexin V binding buffer (cat# 422201, BioLegend, San Diego, CA, USA). A 100 µL aliquot of the cell suspension was transferred into a microcentrifuge tube and stained with 5 µL of APC-conjugated Annexin V (cat# 640920, BioLegend, RRID: AB_2561515) along with 10 µL of PI (Invitrogen, MA, USA). After incubation at room temperature for 15 min in the dark, 400 µL of Annexin V binding buffer was added to each tube. The stained cells were immediately analyzed using a CytoFLEX flow cytometer (Beckman Coulter, Brea, CA, USA, RRID: SCR_019627) to quantify apoptotic populations.

### Immunocytochemistry (ICC)

Cells were washed with PBS and fixed using 4% paraformaldehyde for 15 min on ice. Following three consecutive PBS washes, permeabilization was performed by incubating the cells in a buffer containing 0.2% Triton X-100 in PBS for 10 min at room temperature (RT). To prevent non-specific binding, cells were washed with PBS and treated with a blocking solution composed of 5% bovine serum albumin and 0.3% Triton X-100 in PBS for 20 min at RT. The cells were then incubated with the primary antibody for 2 h at RT. After washing three times with PBS, the cells were exposed to an Alexa Fluor 488-conjugated secondary antibody for 30 min at RT under dark conditions. Cellular mounting was executed using Vectashield mounting medium containing 4′,6-diamidino-2-phenylindole (DAPI; Vector Laboratories, Inc., Burlingame, CA, USA). Fluorescence signals were captured and visualized using a Zeiss 510 fluorescence microscope (Zeiss, Oberkochen, Germany).

### Mitochondrial DNA copy number quantification

To quantify mitochondrial DNA (mtDNA) copy number, total genomic DNA (gDNA) was extracted from the cells using the AccuPrep Genomic DNA Extraction Kit (Cat# K-3032, Bioneer, Daejeon, Republic of Korea) according to the manufacturer’s instructions. The concentration and purity of the extracted gDNA were measured using a NanoDrop spectrophotometer. The relative mtDNA copy number was determined by comparing the levels of the mitochondrial gene ND1 to the nuclear-encoded gene B2M as an endogenous control. Quantitative PCR was performed using PowerUp SYBR Green Master Mix (Cat# A25741, Thermo Fisher Scientific, Waltham, MA, USA) on an ABI StepOne Real-Time PCR System (Applied Biosystems, Warrington, UK). The primer sequences used were as follows: ND1 (Forward: 5’-CCCTAAAACCCGCCACATCT-3’, Reverse: 5’-GAGCGATGGTGAGAGCTAAGGT-3’) and B2M (Forward: 5’-TGCTGTCTCCATGTTTGATGTATCT-3’, Reverse: 5’-TCTCTGCTCCCCACCTCTAAGT-3’). The relative mtDNA copy number was calculated using the 2^−ΔΔct^ method, and the results were normalized to the control group.

### Fluorescence imaging

Mitochondrial morphology fluorescence imaging was performed using Mitotracker Red reagent (cat# M7512, Thermo Fisher scientific). Cells were incubated with 200 nM Mitotracker Red for 30 min at 37 °C. After staining, cells were washed twice with PBS. Subsequently, cells were mounted using Vectashield mounting medium containing DAPI (Vector Laboratories, Inc., Burlingame, CA, USA). Fluorescence images were acquired using a Zeiss 510 fluorescence microscope (Zeiss, Oberkochen, Germany).

### DCFH-DA assay

Intracellular reactive oxygen species (ROS) levels were measured by flow cytometry using the cell-permeant fluorescent probe 2’,7’-dichlorodihydrofluorescein diacetate (DCFH-DA). Following treatment, cells were harvested by trypsinization, washed twice with ice-cold PBS, and collected by centrifugation at 500 × *g* for 5 min. The cell pellets were then resuspended in serum-free medium containing 10 µM DCFH-DA (cat# D399, Thermo Fisher Scientific, Waltham, MA, USA) at a concentration of 1 × 10⁶ cells/mL. After incubation at 37 °C for 30 min in the dark, the cells were washed twice with PBS to remove the extracellular probe and resuspended in 500 µL of PBS. The intensity of DCF fluorescence, which is proportional to the amount of intracellular ROS, was immediately analyzed using a CytoFLEX flow cytometer (Beckman Coulter, Brea, CA, USA) with an excitation wavelength of 488 nm and an emission wavelength of 525 nm. A minimum of 10,000 events were acquired per sample to quantify the mean fluorescence intensity (MFI).

### Quantitative real-time polymerase chain reaction

Total RNA was extracted from cells using TRIzol reagent (cat# 15596026, Ambion, Life Technologies, Carlsbad, CA, USA) following the manufacturer’s instructions. Complementary DNA (cDNA) was synthesized from 1 µg of total RNA using the Takara Prime Script First-Strand cDNA Synthesis kit (cat# 6110 A, Takara Bio, Inc., Shiga, Japan). Quantitative real-time PCR was performed using Power-up SYBR Green Master Mix (cat# A25741, Thermo Fisher Scientific, Waltham, MA, USA) on an ABI StepOne Real-time PCR System (Applied Biosystems, Warrington, UK). Relative gene expression levels were calculated using the 2^−ΔΔct^ method. The primer sequences used were as follows: NQO1 (Forward: 5′-GGCAGAAGAGCACTGATCGTA-3′, Reverse: 5′-TGATGGGATTGAAGTTCATGGC -3′), HMOX1 (Forward: 5′-GGGCCATGAACTTTGTCC-3′, Reverse: 5′-GGCTCCTTCCTCCTTTCC-3′) and GAPDH (Forward: 5′-CAGCCTCAAGATCATCAGCA-3′, Reverse: 5′-TGTGGTCATGAGTCCTTCCA-3′).

### Small interfering RNA (siRNA) transfection

siRNA transfection was performed using Lipofectamine RNAiMAX reagent (cat# 13778-030, Invitrogen, MA, USA) according to the manufacturer’s instructions. Cells were seeded into 60 mm culture dishes and allowed to reach approximately 60–80% confluency at the time of transfection, followed by overnight incubation. SNAIL siRNA (sc-38398, Santa Cruz, Dallas, TX, USA) and Lipofectamine RNAiMAX were each diluted in Opti-MEM® Reduced-Serum Medium (cat# 31985070, Thermo Fisher Scientific), mixed, and incubated at room temperature for 15 min to allow complex formation. The resulting siRNA-lipid complexes were added to the cells, which were subsequently maintained at 37 °C in a humidified CO₂ incubator for 48–72 h. After incubation, cells were collected and lysed, and siRNA transfection efficiency was confirmed by western blotting.

#### PARP response assay

PARP response was assessed as previously described [25]. Whole protein extracts (100 µg) from SNU251-OR and PEO1-OR cells were diluted to a final concentration of 1 µg/µL in PARylation buffer (10 mM HEPES, pH 7.5, 150 mM NaCl, 1 mM MgCl_2_, and 0.5 mM EDTA) and incubated with 1 mM NAD^+^ or IC_50_ concentration of olaparib at 4 °C for 30 min. The reaction was terminated by adding SDS sample buffer, followed by denaturation at 100 °C for 5 min. PARylation level was detected via western blot using an anti-PAR monoclonal antibody (cat# 83732, CST, Danvers, MA, USA, RRID: AB_2749858).

#### Oxygen consumption rate assay

The OXPHOS activity was measured using the Seahorse XF Analyzer (Agilent, RRID: SCR_019545) in conjunction with the XF Cell Mito Stress Test Kit (cat# 103015-100, Agilent), following the manufacturer’s protocol. Briefly, cells were pre-treated for 1.5 h with olaparib, lixumistat, or their combination at IC_50_ concentration before the assay. Then, cells were sequentially treated with Mito Stress Test Kit at defined time intervals. Oligomycin was added to measure ATP-linked respiration. Then, carbonyl cyanide 4-(trifluoromethoxy)phenylhydrazone was used to assess uncoupled mitochondrial respiration and to determine maximal respiratory capacity. Finally, rotenone and antimycin A were utilized to suppress mitochondrial respiration, enabling quantification of non-mitochondrial oxygen consumption rate (OCR). Protein concentration was measured and used to normalize OCR values.

#### NAD^+^/NADH ratio assay

The intracellular NAD^+^/NADH ratio was quantified using a commercial assay kit (cat# 65348, Abcam, Cambridge, UK) according to the manufacturer’s protocol. Briefly, cells were resuspended in 400 μl of NAD^+^/NADH extraction buffer and subjected to two freeze-thaw cycles (at –20 °C for 20 min followed by room temperature for 10 min), with brief vortexing between cycles to ensure efficient lysis. Lysates were then centrifuged at maximum speed at 4 °C for 5 min to remove cell debris, and the supernatant was transferred to a 10 kDa molecular weight spin column (cat# ab93349, abcam) and centrifuged at 10,000 × *g* at 4 °C for 10 min. For NADH-specific detection, 200 μl of the filtrate was incubated at 60 °C for 30 min to selectively decompose NAD^+^. Prepared samples were then mixed with the reaction components supplied in the kit, and absorbance was measured at 450 nm using a microplate reader (Multiskan FC, Thermo Fisher Scientific).

#### Mitochondrial membrane potential assay

Mitochondrial membrane potential (MMP; ΔΨm) was evaluated using the cationic fluorescent dye JC-1 (5,5′,6,6′-tetrachloro-1,1′,3,3′-tetraethylbenzimidazolylcarbocyanine iodide; Dojindo, Kumamoto, Japan). After 24 h of treatment with the indicated drugs, cells were incubated with 2 μM JC-1 dye at 37 °C for 20 min in the dark. Cells were then washed and analyzed using flow cytometry (Beckman Coulter CytoFLEX). Approximately 10,000 cells per sample were examined, and the mitochondrial membrane potential was determined by calculating the ratio of red (aggregated JC-1, –585 nm) to green (monomeric JC-1, –530 nm) fluorescence. Data were analyzed using FlowJo v10 (FlowJo LLC, Oregon, USA, RRID: SCR_008520).

#### Intracellular cytokine staining

Intracellular IL-6 expression was assessed by flow cytometry using the eBioscience™ Foxp3/Transcription Factor Staining Buffer Set (cat# 00-5523-00, Thermo Fisher Scientific). Cells were pre-stained with Fixable Viability Dye (eBioscience), washed, and pelleted at 500 × *g*. The cells were fixed and permeabilized using the Fixation/Permeabilization working solution for 30 min at 4 °C in the dark. After washing twice with 1× Permeabilization Buffer, the cells were resuspended at 1 × 10⁶ cells/mL and stained with FITC-conjugated anti-human IL-6 antibody (cat# 11-7069-82, Thermo Fisher Scientific) for 30 min at 4 °C. The stained cells were washed and analyzed using a CytoFLEX flow cytometer to quantify the IL-6-positive populations.

#### Migration and invasion assay

Migration and invasion assays were performed as previously described [26]. Briefly, cells were seeded into insert wells with serum-free medium and incubated at 37 °C for 24–48 h. For the invasion assay, inserts were pre-coated with 50–100 µL of Matrigel matrix (cat# 254234, Corning, NY, USA) before seeding. After 36 h of incubation, the medium was removed, and unmigrated and non-invaded cells were gently wiped off using cotton swabs. Migrated and invaded cells were then fixed with 2% paraformaldehyde and stained with crystal violet for 5–10 min. Inserts were washed with PBS and distilled water, and then dried for 3–4 h. Cells were visualized under a light microscope, and the migratory and invasive areas were quantified using ImageJ software (version 1.53t, RRID: SCR_003070) by analyzing five randomly selected, non-overlapping fields per insert at ×100 magnification.

#### Cell cycle assay

Cells were cultured in 60 mm culture dishes in complete growth medium at 37 °C. After overnight attachment, cells were treated with the IC_50_ concentration of olaparib and lixumistat for 72 h. Then, cells were harvested and fixed overnight in 70% ethanol at –20 °C. Fixed cells were washed twice with ice-cold PBS, rehydrated, resuspended in RNase A (100 µg/mL), and stained with PI (50 µg/mL) in PBS at room temperature for 20 min. Finally, ~10,000 cells per sample were analyzed using flow cytometry (Beckman Coulter Cytoflex), and cell cycle distribution was calculated based on the proportions of cells in each phase.

#### Establishment of patient-derived tumor xenograft model and development of acquired resistance to olaparib

This animal procedure was performed in accordance with a protocol approved by the Institutional Animal Care and Use Committee (IACUC) of CHA University (IACUC-180124). The establishment of PDTX model was performed as previously described [27]. Briefly, fresh tumor tissues from patients with BRCA-mutated or wild-type ovarian cancer were obtained from the CHA Bundang Medical Center (CHA University, Seongnam, Republic of Korea). These tissues were implanted into four-week-old female NOD.Cg-Prkdc^scid^/J mice to establish F0-PDTX xenografts, which were subsequently serially passaged as F1, F2, F3, and beyond. To develop an acquired olaparib-resistant model (WJO-003-O2), parental PDTX (WJO-003), which harbors BRCA1 mutation, was implanted into 4-week-old female NOD.Cg-Prkdc^scid^Il2rγ^tm1Wjl^/SzJ mice. When tumor volumes reached 80–100 mm^3^, mice were randomized into control (PBS; *N* = 3) or olaparib (*N* = 3). The olaparib group received olaparib via oral gavage at 50 mg/kg once daily for 5 weeks, followed by 50 mg/kg twice daily for 5 weeks, and finally 75 mg/kg twice daily over four months. When tumor volumes exceeded 4000 mm^3^, rapid growing tumor under olaparib treatment was harvested and re-implanted into new NOD.Cg-Prkdc^scid^Il2rγ^tm1Wjl^/SzJ mice (*N* = 3). Olaparib was then administered at 75 mg/kg twice daily and further escalated to 100 mg/kg twice daily. Following previously reported resistance criteria, we slightly modified the definition of resistance to suit our experimental conditions [28]. Tumor exhibiting a rapid growth rate (growth ratio >1.2; based on tumor volume changes over a 2-day interval) was classified as olaparib-resistant and designatedWJO-003-O2.

#### Animal study

This animal procedure was performed in accordance with a protocol approved by the Institutional Animal Care and Use Committee (IACUC) of CHA University (IACUC-200001). Fragments of four ovarian cancer PDTX (CPDX-013, WJO-003, WJO-003-O2, and WJO-013) were implanted subcutaneously into the right flank of 5-week-old female BALB/c-nude mice (JA Bio Inc, Suwon, Korea). When tumor volumes reached 80–100 mm^3^, mice were randomized into four treatment groups, with three to five mice per group (CPDX-013, *n* = 3–4; WJO-003, *n* = 3–4; WJO-003-O2, *n* = 4–5; WJO-013, *n* = 3). The treatment groups were as follows: group 1 (control; PBS, oral gavage, twice daily), group 2 (olaparib, 50 mg/kg, oral gavage, twice daily), group 3 (lixumistat, 45 mg/kg, oral gavage, twice daily), and group 4 (combination of olaparib and lixumistat). All treatments were administered for three weeks, except for the WJO-003 model, which was treated for 34 days. Tumor volumes were measured three times per week and calculated using the formula: tumor length × (tumor width)^2^ × 0.5. Body weight was also monitored three times per week. At the end of the study, all mice, except those designated for the subsequent drug-switching experiment, were euthanized, and tumor tissues were harvested and preserved for further molecular analyses.

#### Drug-switching animal study

To evaluate changes in treatment efficacy between olaparib monotherapy and olaparib combined with lixumistat, we designed an in vivo drug-switching experiment using the WJO-003 and WJO-003-O2 PDTX models. This approach enables direct comparison of treatment effects within the same tumor and genetic background, allowing precise assessment of the impact of adding or withdrawing lixumistat. In the WJO-003 model, on day 35 of initial treatment, mice initially receiving olaparib monotherapy were switched to a combination of olaparib and lixumistat (*N* = 3). Conversely, mice that had been receiving combination therapy were switched to olaparib monotherapy by discontinuing lixumistat (*N* = 3). Treatments continued until day 54. In the WJO-003-O2 model, following the 21-day treatment period, mice in the combination group were switched to olaparib monotherapy (*N* = 1), and treatment was maintained until day 41. Tumor volumes were measured as previously described throughout the study.

### Terminal deoxynucleotidyl transferase dUTP nick end labeling (TUNEL) assay

Apoptotic cells in xenograft tissue sections were detected using a commercial TUNEL staining kit (cat# S7101, Merck, MA, USA) according to the manufacturer’s protocol and as previously described [26]. Tissue slides were deparaffinized, rehydrated through graded alcohols, and subjected to TUNEL staining, followed by counterstaining with hematoxylin. TUNEL-positive cells were quantified by counting five randomly selected, non-overlapping fields per section at ×400 magnification under a light microscope.

### Statistical analysis

All statistical analyses were performed using IBM SPSS Statistics for Windows, version 21 (SPSS, Inc. NY, USA, RRID: SCR_002865). Student’s *t* test was used to compare continuous variables between two independent groups, including cell viability, western blot quantification, apoptosis assay, OCR, NAD^+^/NADH ratio, MMP, migration and invasion, cell cycle distribution, tumor volume, and TUNEL assay. Data were presented as mean ± standard deviation (SD), unless otherwise specified. All *p* values were two-tailed, and values < 0.05 were considered statistically significant.

## Results

### PARP inhibitor-resistant cells are characterized by enhanced PARylation and NAD^+^ production

**Fig. 1:**
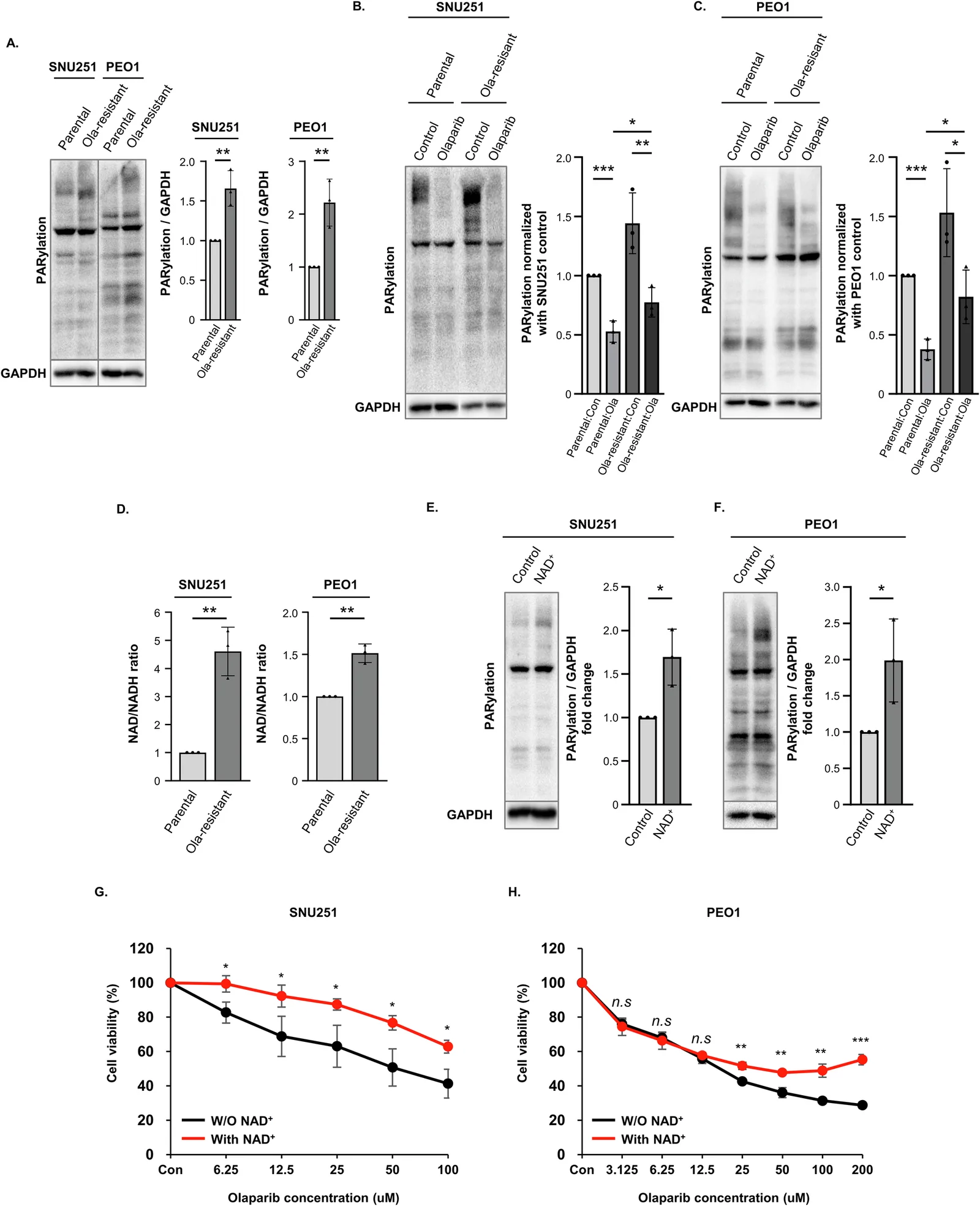
PARP inhibitor-resistant cells are characterized with enhanced PARylation levels and NAD^+^ production. **A** Immunoblot analysis showing the PARylation level in the parental cells (SNU251, PEO1) and olaparib-resistant cells (SNU251-OR, PEO1-OR). Abbreviation: ola: olaparib. Independent-samples *t* test: **p < 0.01. **B**, **C** Immunoblot analysis showing the PARylation response to olaparib in olaparib-resistant cells (SNU251-OR, PEO1-OR) compared to their respective parental cells (SNU251, PEO1) following treatment with the IC_50_ of olaparib determined for the parental cells. Abbreviations: con: control; ola: olaparib. Independent sample *t* test: *p < 0.05, **p < 0.01, ***p < 0.001. **D** NAD^+^/NADH ratio assay in the parental cells (SNU251, PEO1) and olaparib-resistant cells (SNU251-OR, PEO1-OR), respectively. Abbreviation: ola: olaparib. Independent-samples *t* test: **p < 0.01. **E**, **F** Immunoblot assay showing the PARylation level in the parental cells [**E** SNU251, **F** PEO1] following supplementation with exogenous NAD^+^. Independent sample *t* test: *p < 0.05. **G**, **H** Cell viability (MTT) assay of parental cells [**G** SNU251, **H** PEO1] following supplementation with exogenous NAD^+^ and subsequent treatment with the indicated concentration of olaparib for 72 h. Data represent three independent experiments with similar outcomes. W/O without, n.s non-significant. Independent sample *t* test, *p < 0.05, **p < 0.01, ***p < 0.001.

To investigate the underlying mechanisms of PARP inhibitor resistance, we established olaparib-resistant cell lines from BRCA1/2-mutated ovarian cancer cells by gradually increasing the olaparib concentration, starting from 1/100 of parental cells’ IC_50_ [24]. As described in prior studies, PARylation is important for cancer cell survival and stress response pathways. Furthermore, elevated PARylation levels have been associated with resistance to PARP inhibitors [29]. To explore this, we compared the PARylation levels between parental cells (SNU251, PEO1) and olaparib-resistant cells (SNU251-OR, PEO1-OR). Western blot analysis exhibited that basal PARylation levels were significantly increased in the olaparib-resistant cells compared to parental cells (Fig. 1A). Notably, even though the cells were treated with olaparib, olaparib-resistant cells maintained elevated PARylation levels compared to parental cells treated with olaparib (Fig. 1B, C). These results suggest that sustained PARylation levels contribute to the resistance phenotype observed in olaparib-resistant cells.

To investigate the upstream regulatory mechanisms underlying this elevated PARylation, we assessed NAD⁺ production, the essential substrate for PARP1 activity [30]. As expected, the NAD⁺/NADH ratio was significantly increased in olaparib-resistant cells. We hypothesized that enhanced NAD^+^ production supports PARylation (Fig. 1D). To determine whether elevated NAD⁺ levels affect PARylation, we supplemented NAD⁺ to parental cells, resulting in a significant increase in PARylation levels (Fig. 1E, F). Therefore, this indicated that NAD⁺ availability is a limiting factor for PARP activity. Furthermore, supplementation with exogenous NAD⁺ in parental cells reduced their sensitivity to olaparib treatment (Fig. 1G, H). Collectively, these results support that elevated NAD⁺ levels can attenuate the cytotoxic effects of PARP inhibitors by promoting sustained PARylation activity.

### Olaparib-resistant cells enhance OXPHOS activity, and an OXPHOS inhibitor acts synergistically with olaparib

**Fig. 2:**
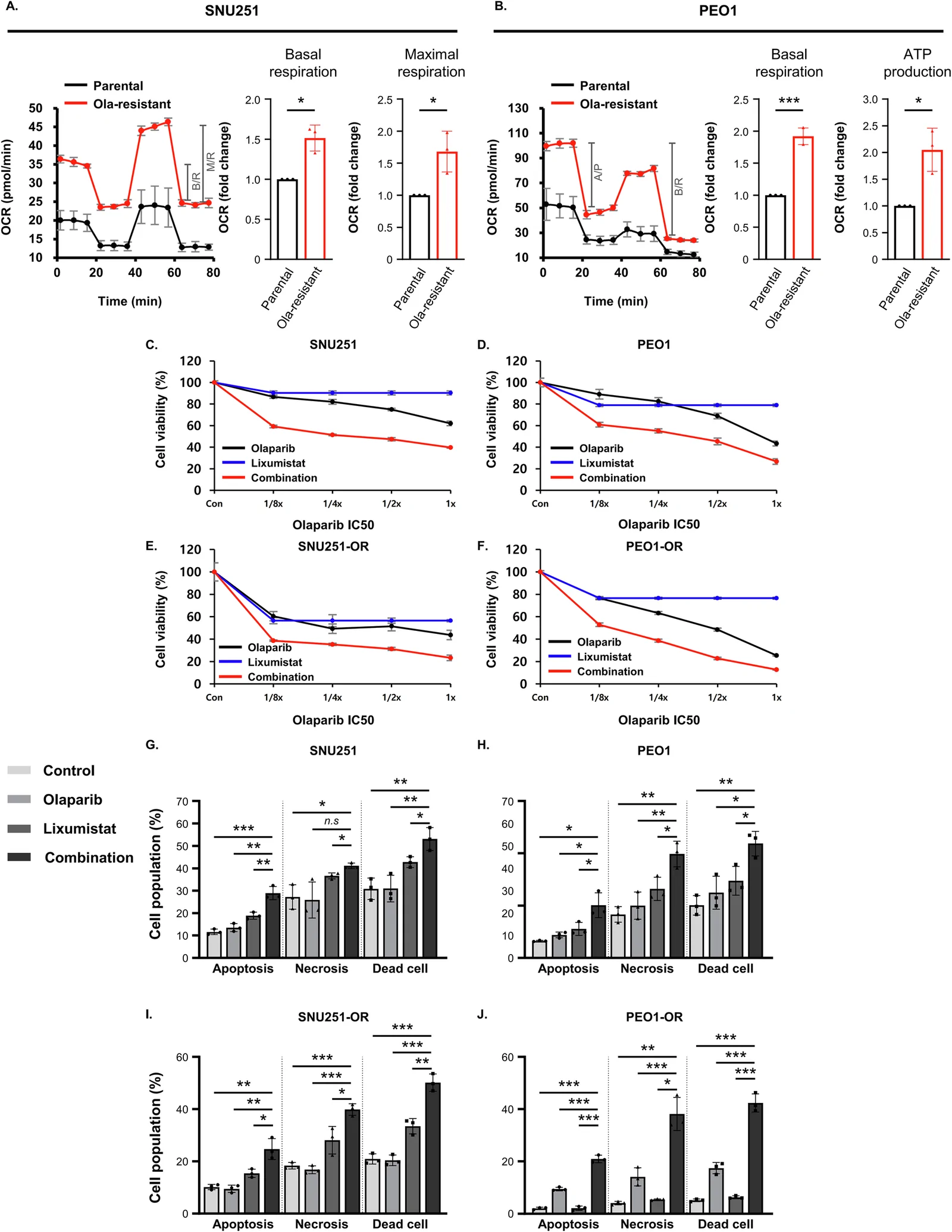
Olaparib-resistant cells increase OXPHOS activity, and OXPHOS inhibitor synergizes with the olaparib. **A**, **B** OCR assay showing OXPHOS activity in the olaparib-resistant cells [**A** SNU251-OR, **B** PEO1-OR] compared with parental cells (SNU251, PEO1), respectively. min: minutes, ola: olaparib; B/R: basal respiration; M/R: maximal respiration; A/P: ATP production. Independent sample *t* test: *p < 0.05, ***p < 0.001. **C**–**F** Cell viability (MTT) assay of parental cells [**C** SNU251, **D** PEO1] and olaparib-resistant cells [**E** SNU251-OR, **F** PEO1-OR] following treatment with olaparib or lixumistat and combination of varying concentrations of olaparib and a fixed concentration of lixumistat for 72 h. Data represent three independent experiments with similar outcomes. **G**–**J** Apoptosis assay demonstrating the synergistic effect of combined olaparib and lixumistat treatment in parental cells [**G** SNU251, **H** PEO1]and olaparib-resistant cells [**I** SNU251-OR, **J** PEO1-OR]. Data are presented as mean ± standard deviation from three independent experiments performed in triplicate. *p* values were calculated by a student *t* test. n.s. non-significant. Independent-sample *t* test, *p < 0.05, **p < 0.01, and ***p < 0.001.

Given the observed increase in PARylation and intracellular NAD⁺ levels in olaparib-resistant cells (SNU251-OR, PEO1-OR), we next sought to elucidate the metabolic mechanisms responsible for NAD⁺ upregulation in these cells. Previous studies have indicated that intracellular NAD⁺ levels can be regulated by multiple pathways, including the NAD⁺ salvage pathway, lactate fermentation, and mitochondrial OXPHOS [31]. However, in this study, gene expression levels of key NAD⁺ salvage pathway enzymes (NAMPT, NMNAT1–3) [32] did not differ significantly between parental cells (SNU251, PEO1) and olaparib-resistant cells (data not shown). Similarly, lactate assay results assessing fermentation-derived NAD⁺ production did not correlate with intracellular NAD⁺ levels (data not shown). We then assessed OXPHOS activity by measuring the OCR. Notably, olaparib-resistant cells consistently exhibited higher OCR levels compared to parental cells, indicating enhanced mitochondrial respiration in the resistant phenotype (Fig. 2A, B). These findings suggest that increased OXPHOS activity may be associated with reduced sensitivity to PARP inhibitors.

To investigate whether OXPHOS inhibition could enhance the efficacy of PARP inhibitors, we conducted proliferation assays in both parental cells and olaparib-resistant (SNU251-OR, PEO1-OR) cells. Combination treatment with OXPHOS inhibitor lixumistat and PARP inhibitor (olaparib or talazoparib) synergistically suppressed cell proliferation compared to OXPHOS inhibitor or PARP inhibitor alone in both sensitive and resistant cell lines, as indicated by CI values < 1 (Fig. 2C–F and Supplementary Fig. 1; Table S2–S3). Even though resistant cells showed a higher IC_50_, the combination treatment was performed with the lower olaparib concentration corresponding to the sensitive setting, rather than arbitrarily increasing the drug dose. As expected, the combination of olaparib and lixumistat at the IC_50_ doses based on the parental cells also exhibited a synergistic effect in the olaparib-resistant cells (CI < 1) (Supplementary Fig. 2; Table S4). Furthermore, apoptosis assays revealed that combined treatment with olaparib and lixumistat significantly increased apoptosis and necrosis compared to monotherapy in both parental and olaparib-resistant cells (Fig. 2G–J). Notably, this cytotoxic effect was specific to cancer cells; in vitro analysis using normal cells (BEAS2B and HEK293T) demonstrated that lixumistat was well-tolerated, showing significantly higher IC_50_ values and no marked induction of apoptosis or necrosis compared to the olaparib-resistant cancer cells (Supplementary Fig. 3).

### Lixumistat overcomes olaparib resistance through inhibiting NAD^+^ production and subsequently suppressing PARylation

**Fig. 3:**
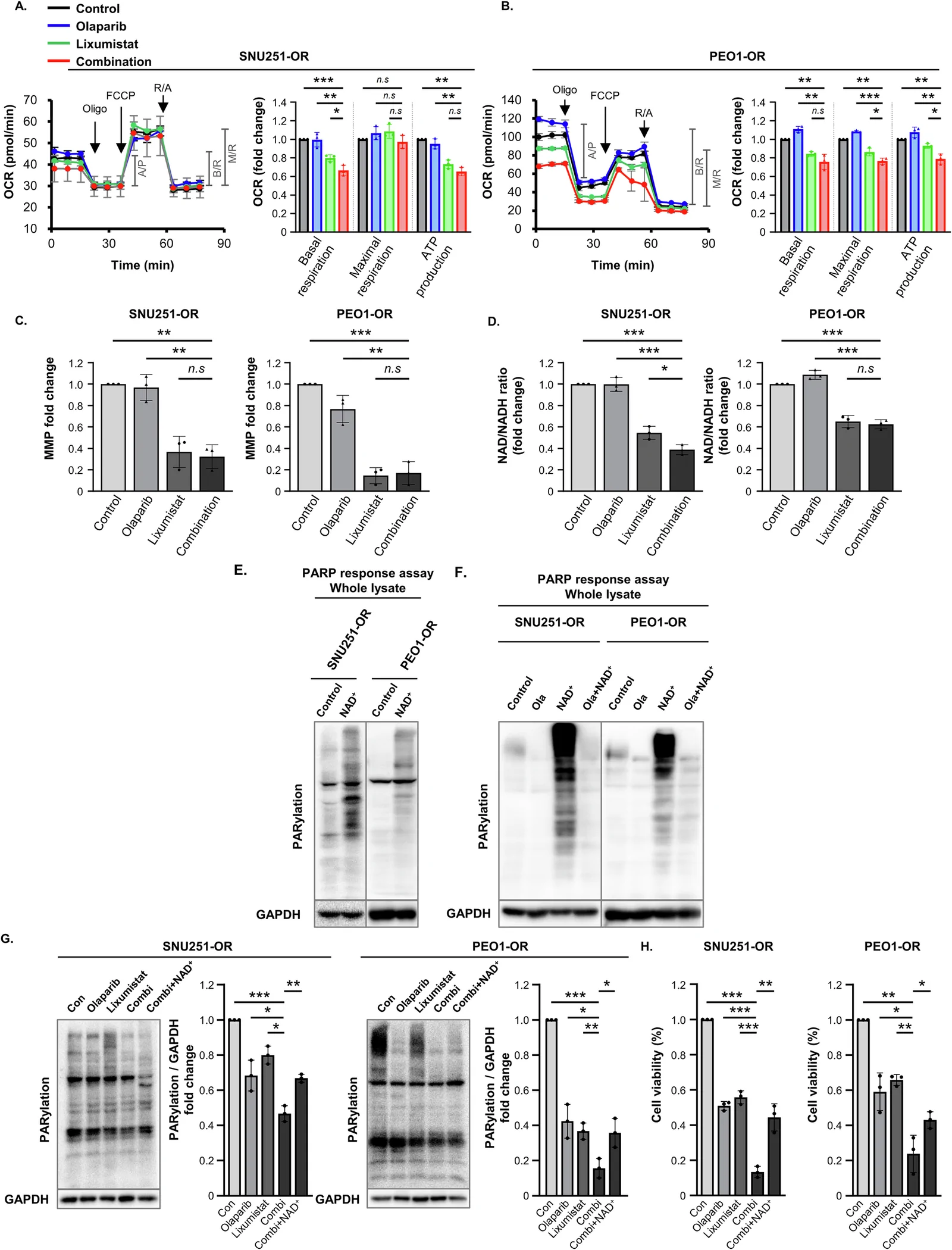
Lixumistat enhances olaparib sensitivity through inhibiting NAD^+^ production and subsequently suppressing PARylation. **A**, **B** OCR assay showing OXPHOS activity in the olaparib-resistant cells [**A** SNU251-OR, **B** PEO1-OR] following treatment of IC_50_ of olaparib and lixumistat (based on the parental cells). R/A rotenone/antimycin A, B/R basal respiration, M/R maximal respiration, A/P ATP production, n.s non-significant, min minutes. Independent sample *t* test, *p < 0.05, **p < 0.01, ***p < 0.001. **C** MMP assay (JC-1) in olaparib-resistant cells (SNU251-OR, PEO1-OR) following treatment with the IC_50_ of olaparib and lixumistat (based on the parental cells). n.s. non-significant. Independent sample *t* test, **p < 0.01, ***p < 0.001. **D** NAD^+^/NADH ratio assay in the olaparib-resistant cells (SNU251-OR, PEO1-OR) following treatment with IC_50_ of olaparib and lixumistat (based on the parental cells). Abbreviation: n.s: non-significant. Independent sample *t* test, *p < 0.05, ***p < 0.001. **E** PARP response assay showing that supplementation with exogenous NAD^+^ to whole lysate increased PARylation levels in the parental cells (SNU251, PEO1). **F** PARP response assay in the olaparib-resistant cells (SNU251-OR, PEO1-OR) following supplementation with exogenous NAD^+^ (1 mM) and IC_50_ of olaparib (based on the parental cells). ola olaparib. **G** Immunoblot analysis of PARylation levels in the olaparib-resistant cells (SNU251-OR, PEO1-OR) following supplementation with exogenous NAD⁺ and the IC_50_ of olaparib and lixumistat (based on the parental cells). combi combination. n.s non-significant. Independent sample *t* test, *p < 0.05, **p < 0.01, ***p < 0.001. **H** Cell viability (MTT) assay of olaparib-resistant cells (SNU251-OR, PEO1-OR) following supplementation with exogenous NAD^+^, followed by treatment with IC_50_ of olaparib and lixumistat (based on the resistant cell lines) for 72 h. Data represent three independent experiments with similar outcomes. combi combination. Independent sample *t* test, *p < 0.05, **p < 0.01, ***p < 0.001.

Based on our findings of elevated OXPHOS activity, increased NAD⁺ production and enhanced PARylation in olaparib-resistant cells (SNU251-OR, PEO1-OR) (Figs. 1A–D and 2A–B), we next investigated whether OXPHOS inhibitor lixumistat could reverse the elevated OXPHOS activity back to baseline levels. To this end, we performed OCR assays in the OXPHOS-activated olaparib-resistant cells. As previously reported, olaparib is a selective PARP inhibitor that primarily targets DNA repair mechanisms, without known direct inhibitory effects on mitochondrial OXPHOS complexes [33, 34]. In contrast, lixumistat treatment, either alone or in combination with olaparib, significantly suppressed OXPHOS activity, including basal respiration, maximal respiration, and ATP production, in olaparib-resistant cells (Fig. 3A, B). Notably, although lixumistat induced a significant decrease in ATP production, exogenous ATP supplementation could not reduce γH2AX levels or restore cell viability (Supplementary Fig. 4), suggesting that ATP exhaustion is not the primary driver of the observed synergistic lethality. Consistent with OXPHOS inhibition, MMP was significantly suppressed following lixumistat treatment, indicating inhibition of mitochondrial function (Fig. 3C). To further characterize the downstream consequences of this functional inhibition, we assessed mitochondrial morphology, ROS production and mitochondrial DNA stability. Lixumistat treatment induced mitochondrial fragmentation, characterized by a granular mitochondrial morphology. Furthermore, lixumistat increased ROS production and decreased mitochondrial DNA copy number (Supplementary Fig. 5). However, our screening of downstream antioxidant markers, such as NRF2, NQO1, and HMOX1, showed inconsistent expression across cell lines, suggesting that ROS-induced oxidative stress may not be the primary driver of the observed synergy (Supplementary Fig. 6).

As described above, NAD⁺ is a critical cofactor in the PARylation process. We examined whether OXPHOS inhibition by lixumistat could alter intracellular NAD⁺ availability. Consistent with the OCR assay results, lixumistat significantly reduced NAD⁺ production, and this suppression was not reversed by combination with olaparib, thereby enhancing the anticancer effects of olaparib in resistant cells (Fig. 3D). These findings suggest that OXPHOS-driven NAD⁺ production supports PARP activity in olaparib-resistant cells. To further validate whether the reduction in NAD⁺ levels is the primary driver of PARP1 functional inhibition, we compared the effects of lixumistat with FK866, a selective NAMPT inhibitor. Similar to lixumistat, FK866 induced a comparable suppression of PARylation in olaparib-resistant cells, and this inhibitory effect was effectively reversed by exogenous NAD⁺ supplementation (Supplementary Fig. 7). To verify whether NAD⁺ levels directly affect PARylation, we performed in vitro PARP response assays. Exogenous NAD⁺ was supplemented to whole cell lysates from olaparib-resistant cells (SNU251-OR and PEO1-OR), leading to a marked increase in PARylation levels in these cells (Fig. 3E). We next evaluated PARP response in whole cell lysates following treatment with olaparib and supplementation with exogenous NAD^+^. As shown in Fig. 3F, exogenous NAD⁺ restored PARylation that had been suppressed by olaparib treatment. Furthermore, when NAD⁺ production was inhibited by lixumistat, PARylation levels were further reduced by the combination of olaparib and lixumistat, indicating a synergistic suppression of PARP activity. Notably, exogenous NAD⁺ supplementation restored the reduced PARylation level induced by the combination treatment (Fig. 3G). Consistent with the PARylation patterns, the combination treatment of olaparib and lixumistat exhibited a synergistic inhibitory effect on cell proliferation, which was restored by NAD^+^ supplementation (Fig. 3H). Taken together, these results suggest that one of the mechanisms by which lixumistat overcomes olaparib resistance is through the inhibition of OXPHOS-dependent NAD⁺ production, thereby limiting substrate availability for PARP1 activity.

### Lixumistat inhibits migratory and invasive behavior and increases olaparib sensitivity

**Fig. 4:**
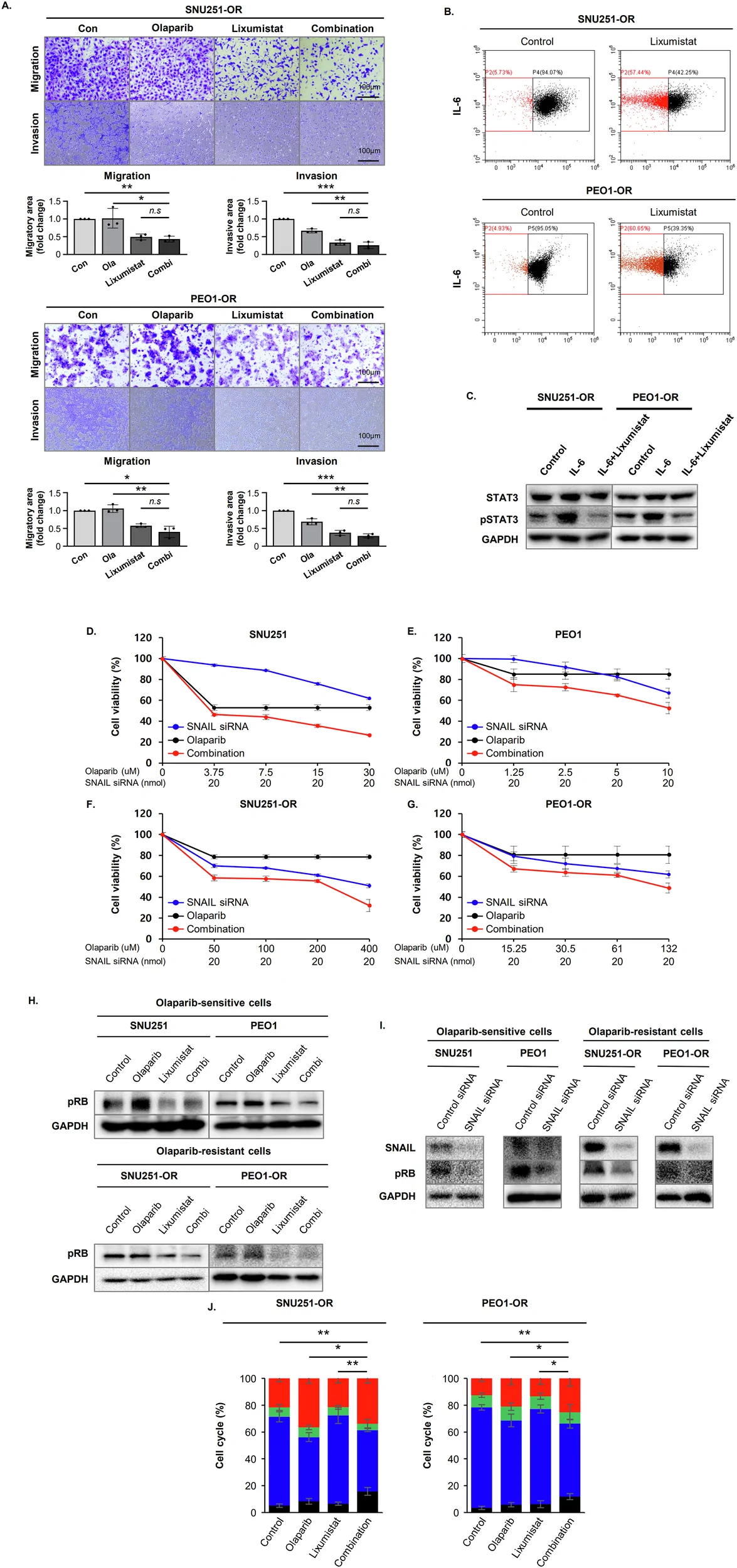
Lixumistat increases olaparib sensitivity via the SNAIL-pRB axis suppression. **A** Representative images from migration and invasion assays of olaparib-resistant cells (SNU251-OR, PEO1-OR) following treatment with the IC_50_ of olaparib and lixumistat (based on the parental cells). Quantification of migratory and invasive areas was performed by analyzing four non-overlapping fields at ×200 magnification, and data are presented as mean ± SD in the bottom panel. ola olaparib, combi combination, n.s non-significant. Independent sample *t* test: *p < 0.05, **p < 0.01, ***p < 0.001. **B** Representative flow cytometry plots of intracellular IL-6 levels in olaparib-resistant cells (SNU251-OR, PEO1-OR) following lixumistat treatment. Cell populations were gated to distinguish between IL-6-negative (red box) and IL-6-positive (black box) cells. **C** Immunoblot analysis of STAT3 and pSTAT3 levels in the olaparib-resistant cells (SNU251-OR, PEO1-OR) following treatment with IL-6 and lixumistat. **D**–**G** Cell viability (MTT) assay of the parental cells [**D** SNU251, **E** PEO1] and olaparib-resistant cells [**F** SNU251-OR, **G** PEO1-OR] following treatment with SNAIL siRNA or olaparib and a combination of varying concentrations of olaparib and a fixed concentration of siRNA for 72 h. Data represent three independent experiments with similar outcomes. **H** Immunoblot analysis of pRB expression in parental cells (SNU251, PEO1) and olaparib-resistant cells (SNU251-OR, PEO1-OR) following treatment with the IC_50_ of olaparib and lixumistat (based on the parental cells). combi combination. **I** Immunoblot analysis of pRB expression following SNAIL knockdown using SNAIL siRNA in parental cells (SNU251, PEO1) and olaparib-resistant cells (SNU251-OR, PEO1-OR). **J** Cell cycle analysis by PI staining in olaparib-resistant cells (SNU251-OR, PEO1-OR) following treatment with the IC_50_ of olaparib and lixumistat (based on the parental cells). The bar graph represents the distribution of cell populations across different phases of the cell cycle. Independent-sample *t* test: *p < 0.05, **p < 0.01.

Previous studies have demonstrated that biguanides such as metformin can interfere with epithelial–mesenchymal transition (EMT) [35, 36]. Based on this, we investigated whether lixumistat affects migratory and invasive behaviors in both parental cells (SNU251, PEO1) and olaparib-resistant cells (SNU251-OR, PEO1-OR) following treatment with the indicated compounds. Lixumistat treatment significantly suppressed the migratory and invasive capacities of both parental and olaparib-resistant cells, and this effect was further enhanced when combined with olaparib (Fig. 4A and Supplementary Fig. 8). To explore the underlying mechanisms, we assessed the expression of EMT markers. Although the expression pattern of these markers varied across four cell lines, SNAIL expression was consistently suppressed by lixumistat, either alone or in combination with olaparib (Supplementary Fig. 9A). Accordingly, we hypothesized that SNAIL could serve as a potential key regulator of both PARP inhibitor sensitivity and migratory behavior in ovarian cancer cells. Consistent with lixumistat treatment, SNAIL knockdown markedly reduced migratory and invasive behavior in both parental and olaparib-resistant cells, supporting that the anti-migratory effects of lixumistat were regulated through downregulation of SNAIL (Supplementary Fig. 9B). To further elucidate the downstream signaling pathway through which SNAIL mediates this migratory and invasive behavior, we screened various cytokines and observed a consistent reduction in IL-6 and pSTAT3 levels following lixumistat treatment (Fig. 4B, C). To determine whether this regulation is SNAIL-dependent, we found that while exogenous IL-6 promoted migratory behaviors, it did not alter SNAIL expression (Supplementary Fig. 9C, D). In contrast, SNAIL knockdown significantly reduced both intracellular IL-6 and pSTAT3 levels, indicating that SNAIL acts as a critical upstream regulator of the IL-6/pSTAT3 axis (Supplementary Fig. 9E, F). Additionally, SNAIL is widely reported as a master regulator of EMT in ovarian cancer; emerging evidence also implicates it in regulating cell survival and drug sensitivity [37, 38]. To determine whether SNAIL affects olaparib sensitivity, we conducted cell proliferation assays in both parental cells and olaparib-resistant cells following treatment with SNAIL knockdown, olaparib, or their combination. As expected, the combination of SNAIL knockdown and olaparib resulted in a synergistic reduction in cell proliferation in both parental and olaparib-resistant cells, as indicated by combination index (CI) values < 1 (Fig. 4D–G; Table S5). In contrast, combination treatment with control siRNA and olaparib did not exhibit synergy (CI > 1) (Supplementary Fig. 10; Table S6).

To further investigate the downstream mechanisms involved in SNAIL-mediated olaparib sensitivity, we examined potential candidate regulators (data not shown). Several potential regulators were considered, including BCL2 [39], p21 [40], p53 [41], PTEN [42] and phosphorylated retinoblastoma (pRB) [37]. Among these markers, only pRB showed consistent downregulation by lixumistat treatment and SNAIL knockdown (Fig. 4H, I; other data not shown). Finally, cell cycle analysis revealed that combination treatment of lixumistat and olaparib led to a significant increase in the SubG1 population, indicating enhanced DNA damage and apoptosis (Fig. 4J). Collectively, these findings suggest that lixumistat enhances olaparib sensitivity through inhibition of the SNAIL-pRB axis. Furthermore, this regulatory axis increased apoptosis in both parental and olaparib-resistant cells. Therefore, this highlights the broader therapeutic potential of lixumistat as a PARP inhibitor sensitizer.

### Combined treatment with olaparib and lixumistat synergistically suppresses both olaparib-sensitive and -resistant ovarian cancer in PDTX models

**Fig. 5:**
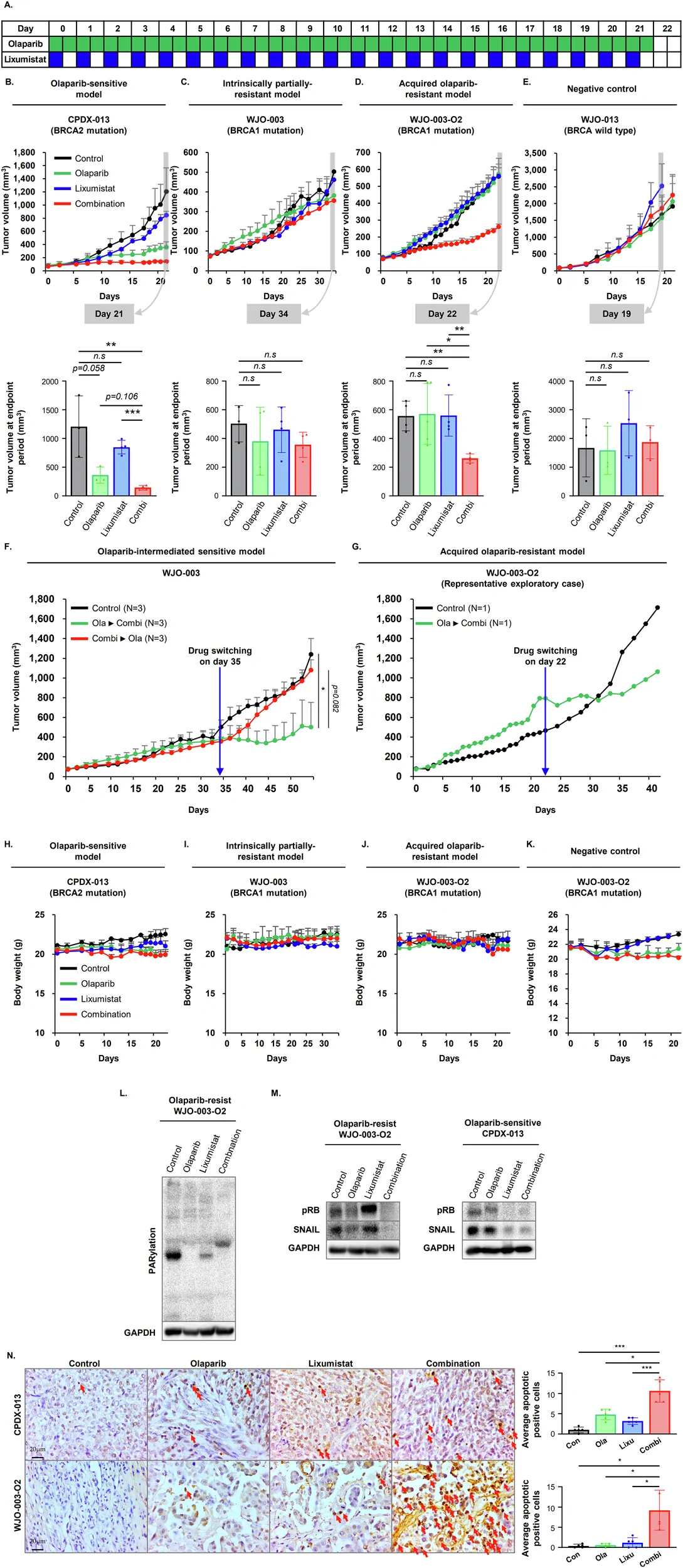
Combined treatment of olaparib and lixumistat synergistically suppresses both olaparib-sensitive and -resistant ovarian cancer in PDTX models. **A** Experimental design for in vivo efficacy test using olaparib-sensitive and -resistant PDTX models in BALB/c nude mice. When tumor volumes reached 60–80 mm^3^, mice were randomized to control, olaparib, lixumistat, or combination groups. Each group included 3–5 mice per model, with specific allocations as follows: CPDX-013 (*n* = 3, 4), WJO-003 (*n* = 3, 4), WJO-003-O2 (*n* = 4, 5), WJO-013 (*n* = 3). Olaparib (50 mg/kg) was given via oral gavage twice daily for 3 weeks. Lixumistat (45 mg/kg) was given via oral gavage once daily for 3 weeks. **B**–**E** Average tumor growth curves for (**B**) CPDX-013 (BRCA2-mutated, “olaparib-sensitive”), **C** WJO-003 (BRCA1-mutated, “intrinsically partially-resistant”), **D** WJO-003-O2 (BRCA1-mutated, “acquired olaparib-resistant”), and **E** WJO-013 (BRCA wild-type, “olaparib-unresponsive”, negative control) PDTX models were treated with the indicated compounds. The bar graphs illustrate the average tumor volumes of the indicated treatment groups at the end of the initial treatment period. Mice were euthanized and tumors collected 22 days after treatment initiation (day 34 for WJO-003). Error bars represent SEM of 3–4 mice per group. combi combination, n.s. non-significant. Independent sample *t* test, *p < 0.05, **p < 0.01, ***p < 0.001. **F** Average tumor growth in the WJO-003 model (intrinsically partially-resistant) under a treatment-switching schedule. In the initial olaparib group, treatment was switched to the combination of olaparib and lixumistat starting on day 35. In the initial combination group, treatment was switched to olaparib monotherapy starting on day 35. Abbreviations: ola: olaparib; combi: combination. Independent sample *t* test, *p < 0.05. **G** Individual tumor growth in the WJO-003-O2 model (acquired olaparib-resistant) under a treatment-switching schedule. In the initial olaparib group, treatment was switched to the combination of olaparib and lixumistat starting on day 22. In the initial combination group, treatment was switched to olaparib monotherapy starting on day 22. ola olaparib, combi combination. **H**–**K** Average body weight of **H** CPDX-013, **I** WJO-003, **J** WJO-003-O2, **K** WJO-013. Error bars represent SEM of 3–4 mice per group. **L** Immunoblot analysis of WJO-003-O2 tumor samples showing suppression of PARylation in the combination treatment group. **M** Immunoblot analysis of WJO-003-O2 and CPDX-013 tumor samples showing suppression of SNAIL and pRB in the lixumistat and combination treatment groups. **N** TUNEL assay was performed on PDTX tumor tissues collected at the time of sacrifice. Representative staining images for each treatment group are shown. Bar graphs depict the average number of apoptotic cells per group, quantified from five random, non-overlapping fields at ×400 magnification. Data are presented as mean ± SD. con control, ola olaparib, lixu lixumistat, combi combination. Independent sample *t* test: *p < 0.05, ***p < 0.001.

Based on our in vitro findings, we next investigated whether lixumistat, alone or in combination with olaparib, could inhibit tumor growth in vivo. To this end, we established four distinct ovarian cancer PDTX models: CPDX-013 (BRCA2-mutated, hereafter “olaparib-sensitive”), WJO-003 (BRCA1-mutated, “intrinsically partially-resistant”), WJO-003-O2 (BRCA1-mutated, “acquired olaparib-resistant”), and WJO-013 (BRCA wild-type, “olaparib-unresponsive”, negative control). When tumor volumes reached 60–80 mm³, mice were treated with olaparib (oral gavage, twice daily) and lixumistat (once daily) for 3 weeks. Mice were euthanized and tumors were collected 22 days after treatment initiation (day 34 for WJO-003) (Fig. 5A).

In the CPDX-013 model, olaparib monotherapy remarkably reduced tumor growth, confirming its classification as olaparib-sensitive. Notably, combined treatment with lixumistat further enhanced antitumor efficacy (Fig. 5B and Supplementary Fig. 11; Table S7). In contrast, the WJO-003 model exhibited limited response to short-term olaparib treatment (30 days), but demonstrated delayed antitumor activity with long-term exposure (up to 90 days) in an independent study (Supplementary Fig. 12), supporting its characterization as intrinsically partially resistant. Consistently, in the present study, WJO-003 did not respond to any treatment, including olaparib monotherapy, until day 34 (Fig. 5C and Supplementary Fig. 13; Table S8). In the acquired-resistant WJO-003-O2 model, significant tumor suppression was observed only in the combination treatment group, with neither olaparib nor lixumistat alone showing efficacy (*p* < 0.05) (Fig. 5D and Supplementary Fig. 14; Table S9). Finally, the BRCA wild-type WJO-013 model was unresponsive to olaparib, and no treatment group—including the combination—exhibited antitumor activity (Fig. 5E and Supplementary Fig. 15; Table S10).

We next conducted drug-switching experiments in the WJO-003 (olaparib-unresponsive) and WJO-003-O2 (acquired olaparib-resistant) models, transitioning from monotherapy to combination treatment and vice versa. In the WJO-003 model, which had previously shown no response to any treatment, tumor growth was significantly suppressed after switching from olaparib monotherapy to the combination therapy (*p* < 0.05). In contrast, switching from the combination to olaparib alone resulted in rapid tumor regrowth, similar to the control group (Fig. 5F). Likewise, in the WJO-003-O2 model (representative exploratory case; *N* = 1), which exhibited acquired resistance to olaparib, tumor growth was dramatically reduced following the switch from olaparib monotherapy to combination treatment (Fig. 5G). Although this experiment was performed as a pilot exploratory study with only a single mouse per group in the WJO-003-O2 model, the observed response pattern mirrored that of the WJO-003 model. While we acknowledge the limitation of the sample size in the WJO-003-O2 model (*N* = 1), these findings suggest that the synergistic effect is not an incidental artifact but rather a fundamental mechanistic requirement for overcoming acquired PARP inhibitor resistance, further supporting the potential benefit of early or adaptive integration of lixumistat. Furthermore, none of the treatments caused weight loss, indicating a lack of generalized toxicity (Fig. 5H–K).

Subsequently, tumor tissues excised from the PDTX models were subjected to molecular analysis. Consistent with our in vitro findings, western blot analysis of the olaparib-resistant tumors (WJO-003-O2) exhibited dual inhibition of PARylation and SNAIL-pRB expression in the combination group, compared to either olaparib or lixumistat monotherapy. In addition, olaparib-sensitive tumors (CPDX-013) showed inhibition of SNAIL-pRB expression (Fig. 5L, M). TUNEL staining of xenograft tumors demonstrated that the combination of olaparib and lixumistat significantly increased apoptotic activity, compared to monotherapy in both olaparib-sensitive (CPDX-013) and olaparib-resistant (WJO-003-O2) models (*p* < 0.05; Fig. 5N).

### Schematic illustration of dual mechanisms by which OXPHOS inhibition enhances PARP inhibitor efficacy

**Fig. 6:**
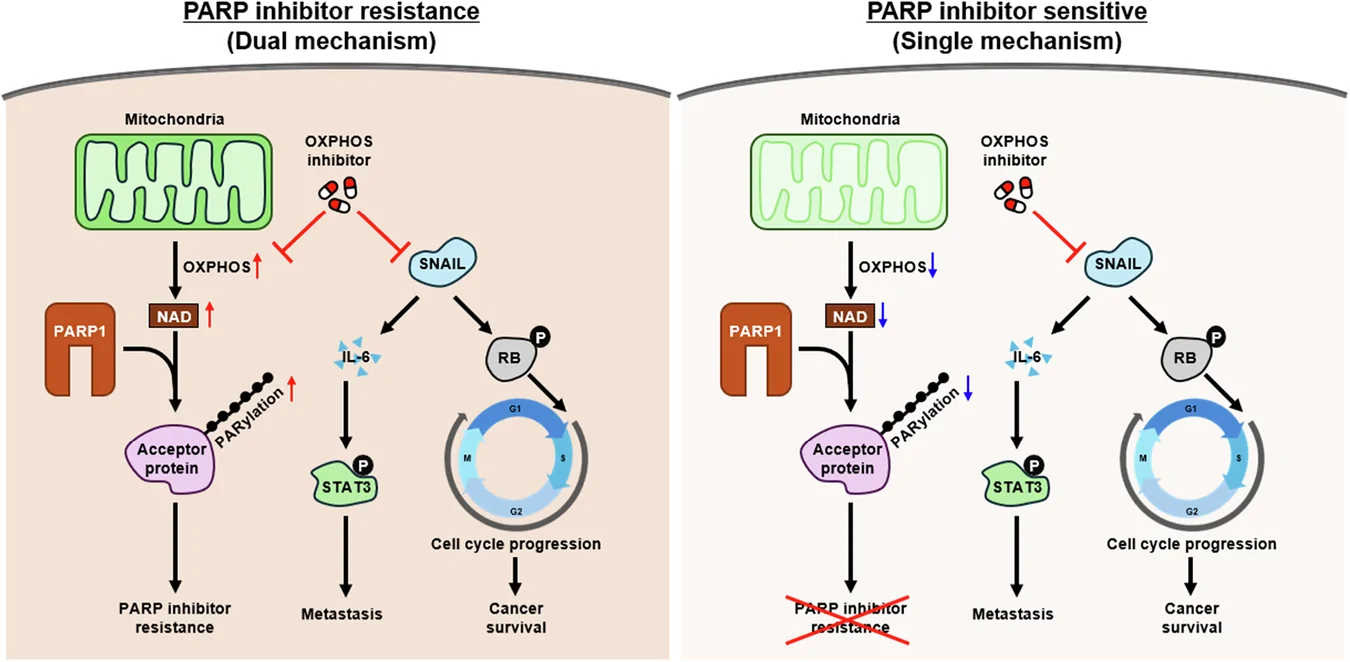
Schematic diagram showing the proposed mechanism by which OXPHOS inhibition contributes to PARP inhibitor resistance. In PARP inhibitor-resistant cancer (left panel), OXPHOS activity is elevated, leading to increased NAD+ production, thereby enhancing PARylation. Furthermore, OXPHOS inhibition suppresses the SNAIL–IL6–pSTAT3 and pRB axes, thereby increasing PARP inhibitor sensitivity and inhibiting metastatic activity. On the contrary (right panel), in the PARP inhibitor-sensitive cancer (right panel), where OXPHOS activity is low, OXPHOS inhibition primarily affects the SNAIL–IL6–pSTAT3/pRB axis, resulting in increased PARP inhibitor sensitivity and suppression of metastasis.

As illustrated in Fig. 6, OXPHOS inhibition enhances the efficacy of PARP inhibitors via distinct mechanisms depending on cellular OXPHOS activity. In olaparib-resistant cells, characterized by elevated OXPHOS activity, inhibition of the OXPHOS–NAD⁺–PARylation axis limits NAD⁺ availability for PARP1, thereby reducing PARylation and restoring PARP inhibitor sensitivity. Concurrently, suppression of the SNAIL–IL6–pSTAT3 and SNAIL–pRB axis impairs metastatic potential and cell survival. In contrast, olaparib-sensitive cells with low baseline OXPHOS activity are unaffected in terms of the PARylation pathway. However, OXPHOS inhibition still attenuates the SNAIL–mediated IL6–pSTAT3 and pRB signaling, contributing to reduced metastasis and survival, and thereby augmenting the cytotoxic effect of PARP inhibitors. Thus, regardless of PARP inhibitor sensitivity, OXPHOS blockade potentiates PARP inhibitor efficacy through shared and distinct mechanisms across both olaparib-sensitive and -resistant tumors.

## Discussion

This study assessed the novel biguanide lixumistat combined with PARP inhibition in olaparib-sensitive and -resistant BRCA1/2-mutated ovarian cancer models. Given that up to 18% of ovarian cancers harbor BRCA mutations and that 40–50% of patients develop resistance to PARP inhibitors, novel strategies are needed. Here, we demonstrate for the first time that lixumistat restored olaparib activity in olaparib-resistant preclinical models by inhibiting the OXPHOS-NAD⁺-PARylation axis, and suppressing SNAIL–pRB axis. Collectively, lixumistat enhanced olaparib activity in olaparib-sensitive preclinical models, suggesting that adding lixumistat to PARP inhibitors is a promising strategy for both PARP inhibitor-naive and -resistant ovarian cancer.

Our study demonstrated a novel mechanism of PARP inhibitor resistance, wherein enhanced OXPHOS activity contributes to PARP inhibitor resistance. Olaparib-resistant BRCA1/2-mutated ovarian cancer cells exhibited significantly higher OCR than their parental counterparts. Although OXPHOS blockade has been proposed as a combination partner for PARP inhibitors based on enhanced activity [23], a direct mechanistic link between metabolic reprogramming and PARP inhibitor resistance had not been established [13]. Up-regulated OXPHOS was reported to elevate NAD⁺, the essential substrate for PARP1, thereby sustaining PARylation that promotes DNA repair, cell-cycle progression, oncogenic metabolic reprogramming, and oncogenic signaling [43, 44]. In line with previous reports, we demonstrated that lixumistat, a novel biguanide, curtailed NAD⁺ production, suppressed PARylation, and restored olaparib sensitivity. Our data demonstrate that the synergistic lethality of lixumistat is primarily driven by a metabolic crisis via NAD^+^ depletion, rather than by oxidative stress or ATP exhaustion. While lixumistat increases ROS, the inconsistent antioxidant response suggests that oxidative stress is a secondary effect. These differences between the two cell lines are likely due to their distinct genetic backgrounds and baseline antioxidant capacities. For example, different baseline levels of the NRF2 pathway or variations in how each cell line responds to oxidative stress can cause these secondary markers to change differently after drug treatment. Importantly, however, despite these varying responses in the ROS/NRF2 axis, our main target pathways, the OXPHOS-NAD⁺-PARylation axis and the SNAIL-pRB pathway, were equally and consistently suppressed in both cells. This clearly indicates that the metabolic crisis, rather than oxidative stress, is the true universal mechanism of lixumistat. Furthermore, the failure of exogenous ATP to rescue cell viability confirms that this synergy is independent of general energy depletion, identifying the disruption of the OXPHOS-NAD^+^-PARylation axis as the critical determinant of cell death in olaparib-resistant cells. Additionally, lixumistat also reduced SNAIL expression, lowering RB phosphorylation, impairing cell-cycle progression, and consequently inducing apoptosis. Thus, lixumistat overcame PARP inhibitor resistance by targeting the OXPHOS-NAD⁺-PARylation axis and, independently, by inhibiting the SNAIL–pRB pathway.

In our drug-switching mouse experiments, both the intrinsically partial-resistant model and the acquired-resistant model showed limited response to olaparib monotherapy. However, a marked tumor suppression was observed upon switching to combined lixumistat and olaparib therapy. These findings suggest that early integration of OXPHOS inhibition may be more effective in preventing or delaying resistance, rather than relying on salvage strategies after resistance has emerged. Clinically, this provides a rationale for front-loaded or adaptive combination approaches in high-risk ovarian cancer patients receiving maintenance PARP inhibitor therapy.

To speculate on the potential toxicities of lixumistat, we reviewed data on OXPHOS inhibition. Mitochondrial OXPHOS dysfunction has been linked to severe fetal outcomes—intrauterine growth restriction, abnormal amniotic fluid, and preterm birth—and knockout of key OXPHOS genes (e.g., Tfam, PolgA, Ndufs4, Cox15, Drp1, Mfn2) causes embryonic lethality or major malformations [45], indicating that pharmacological OXPHOS blockade could pose pregnancy risks. Furthermore, it is known that PARP1 overactivation and NAD⁺ depletion can lead to multi-organ damage [46, 47]. In our mouse study, however, no weight loss occurred, suggesting limited systemic toxicity in adults. The phase I clinical data of lixumistat from NCT03272256 also demonstrated a manageable safety profile without fatal systemic toxicity. The most frequent treatment-related adverse events were primarily gastrointestinal, such as nausea (73%), diarrhea (55%), and vomiting (50%), which were generally transient and reversible [48]. These clinical findings suggest that the level of NAD⁺ depletion induced by lixumistat monotherapy is effectively tolerated at clinically relevant doses. However, PARP inhibitors also cause nausea, vomiting, and myelosuppression [49]. Therefore, considering that both lixumistat and PARP inhibitors impact cellular energy metabolism and DNA repair mechanisms, the potential for augmented systemic toxicity during combination therapy must be carefully monitored in future clinical investigations to ensure patient safety.

Although many preclinical studies have explored strategies to enhance PARP inhibitor efficacy, only a few drugs have progressed to clinical trials. There are several ongoing or completed trials to investigate combinations targeting oncogenic signaling (e.g., VEGF, PI3K-Akt, mTOR) [50, 51], immune checkpoints (PD-1/PD-L1) [52], DNA damage response (ATR, WEE1) [53, 54], and cell cycle regulators (CDK, CHK1) [51, 55] with PARP inhibitors. Among these, the phase III PAOLA-1 trial demonstrated that the combination of olaparib with bevacizumab significantly improved progression-free survival in patients with advanced ovarian cancer [50]. In addition, pembrolizumab combined with niraparib showed modest efficacy (overall response rate 18%) in patients with platinum-resistant ovarian cancer [52]. Despite these advances, an unmet need remains. Our study presents a novel approach targeting the OXPHOS pathway, demonstrating that lixumistat synergizes with PARP inhibitors. Notably, lixumistat is orally bioavailable and well-tolerated, making it a practical and promising combinatorial partner for oral PARP inhibitor-based therapies.

Our findings have important implications for clinical translation. The identification of OXPHOS activation as a driver of PARP inhibitor resistance suggests its potential as a predictive biomarker for patient selection, enabling identification of patients who may benefit from combined OXPHOS and PARP inhibition. These results provide a strong rationale for clinical trials evaluating PARP inhibitors with OXPHOS inhibitors such as lixumistat in BRCA-mutated, OXPHOS-activated ovarian cancer. Such strategies may initially target patients with PARP inhibitor-resistant disease and later be extended to PARP inhibitor-naive patients in the maintenance setting.

This study has several limitations. First, the lack of validation in human clinical samples restricts the translational relevance of our findings. In the future, tumor tissues from patients with BRCA-mutated ovarian cancer should be collected to detect the correlation between OXPHOS activity and the efficacy of PARP inhibitors, thereby verifying the clinical applicability of our proposed mechanism. Second, while lixumistat demonstrated promising efficacy in overcoming PARP inhibitor resistance, its potential off-target effects require further investigation due to its broad metabolic impact. Finally, lixumistat was tested at a single fixed dose in our in vivo studies without dose-response or pharmacokinetic assessment. To address this, subsequent dose-effect experiments combined with pharmacokinetic analysis are planned to determine the optimal clinical dosage and to refine clinical dosing strategies.

In conclusion, this study demonstrated that enhanced OXPHOS activity is a novel mechanism underlying PARP inhibitor resistance in BRCA1/2-mutated ovarian cancer. Our study validated that lixumistat, a selective OXPHOS inhibitor, synergistically enhances the efficacy of PARP inhibitors in both olaparib-naive and -resistant preclinical PDTX models. Importantly, early combination of a PARP inhibitor with lixumistat may boost anticancer activity and delay the acquisition of PARP inhibitor resistance. These findings support lixumistat as a promising therapeutic partner for PARP inhibitors in PARP inhibitor-naive and -resistant ovarian cancer patients.

## Supplementary information

Supplementary Material

Supplementary Material - Original western blot

## Data Availability

All the data supporting the findings of this study are available within the paper and its Supplementary Information files. All other data supporting the findings of this study are available from the corresponding author upon reasonable request.
