# Supplementary material for: Targeting oxidative phosphorylation with lixumistat overcomes PARP inhibitor resistance in ovarian cancer
Source: Cell Death Dis. 2026 Aug 7;17(1):939. doi: 10.1038/s41419-026-09140-y (PMC13737184; doi:10.1038/s41419-026-09140-y)
Supplement: Supplementary file 1 [file 41419_2026_9140_MOESM1_ESM.docx]

**Figure S1. Lixumistat enhances talazoparib sensitivity**

A-D. Cell viability (MTT) assay of parental cells [(A) SNU251, (B) PEO1] and olaparib-resistant cells [(C) SNU251-OR, (D) PEO1-OR] following treatment with talazoparib or lixumistat and combination of varying concentrations of talazoparib and a fixed concentration of lixumistat for 72 h. Data represent three independent experiments with similar outcomes.

**Figure S2. Lixumistat enhances olaparib sensitivity in olaparib-resistant cells**

A-B. Cell viability (MTT) assay of olaparib-resistant cells [(A) SNU251-OR, (B) PEO1-OR] following treatment with olaparib or lixumistat and combination of varying concentrations of lixumistat and a fixed IC_50_ of olaparib and lixumistat (based on the parental cells) for 72 h. Data represent three independent experiments with similar outcomes.

**Figure S3. Selective cytotoxicity of lixumistat in ovarian cancer cells versus normal cells**

A. Cell viability (MTT) assay of normal cell (BEAS2B, HEK293T) and olaparib-resistant cells (SNU251-OR, PEO1-OR) following treatment with indicated concentrations of lixumistat for 72 h. Data represent three independent experiments with similar outcomes.

B. Apoptosis assay demonstrates the apoptosis and necrosis in normal cell (BEAS2B, HEK293T) and olaparib-resistant cells (SNU251-OR, PEO1-OR) following treatment with same concentration of lixumistat for 24 h. The bar graphs represent the percentage of apoptotic, necrotic, and total dead cells normalized to the control group. Data are presented as mean ± standard deviation from three independent experiments performed in triplicate.

**Figure S4. Lixumistat-induced cytotoxicity and DNA damage are independent of intracellular ATP depletion.**

A. Cell viability (MTT) assay of olaparib-resistant cells (SNU251-OR, PEO1-OR) following supplementation with exogenous ATP, followed by treatment with half of the IC_50_ of olaparib and lixumistat (based on the resistant cell lines). Data represent three independent experiments with similar outcomes. Abbreviations: con: control; ola: olaparib; lixu: lixumistat; combi: combination; n.s: non-significant

B. Immunofluorescence images showed the ɣH2AX foci formation in olaparib-resistant cells (SNU251-OR, PEO1-OR) following supplementation with exogenous ATP, followed by treatment with IC_50_ of olaparib and lixumistat (based on the resistant cell lines). The images were taken at 100x magnification. The bar graphs depicted the average foci number per cell. Data were averaged from three independent experiments, with 100 cells analyzed per condition in each experiment. Data are presented as mean ± SD. *P* values were calculated by Student’s *t*-test, indicating ***p* < 0.01, ****p* < 0.001. Abbreviations: con: control; ola: olaparib; lixu: lixumistat; combi: combination; n.s: non-significant.

**Figure S5. Functional and structural analysis of mitochondria following lixumistat treatment**

A. Immunofluorescence images showed the mitochondrial morphology in SNU251-OR and PEO1-OR cells. Mitochondria were stained with MitoTracker Red (red), and nuclei were counterstained with DAPI (blue). Cells were treated with IC50 of lixumistat for 24 h. The images were taken at 40-400x magnification (Scale bar: 50 µm for SNU251-OR, 10 µm for PEO1-OR).

B. Relative reactive oxygen species (ROS) levels were detected using the DCFH-DA fluorescent probe in SNU251-OR and PEO1-OR cells following treatment with IC50 of lixumistat for 12 h. The bar graphs depict the fold change in ROS production relative to the control group. Data represent the mean ± SD from three independent experiments. Independent sample t-test: ***p* < 0.01, ****p* < 0.001.

C. The ratio of mitochondrial DNA (ND1) to nuclear DNA (B2m) was determined by real-time quantitative PCR in SNU251-OR and PEO1-OR cells following treatment with IC50 of lixumistat for 24 h. The bar graphs depict the fold change in ROS production relative to the control group. Data represent the mean ± SD from three independent experiments. Independent sample t-test: ****p* < 0.001.

**Figure S6. Comparison of ROS and antioxidant markers between olaparib-resistant cells and normal cells**

A. DCFH-DA (ROS) assay of olaparib-resistant cells (SNU251-OR, PEO1-OR) following treatment with the IC_50_ of olaparib and lixumistat (based on the parental cells). Data were presented as mean ± SD of triplicate experiments.

B. Immunoblot analysis of NRF2 levels in the olaparib-resistant cells (SNU251-OR, PEO1-OR) following treatment with IC_50_ of olaparib and lixumistat (based on the parental cells).

C. The relative mRNA expression of NRF2 and HMOX1 were determined by qRT-PCR in the olaparib-resistant cells (SNU251-OR, PEO1-OR) following treatment with IC_50_ of olaparib and lixumistat (based on the parental cells). Data were presented as mean ± SD of triplicate experiments.

**Figure S7. Comparison of PARylation suppression between lixumistat and the NAMPT inhibitor FK866 in olaparib-resistant cells**

Immunoblot analysis of PARylation levels in olaparib-resistant cells (SNU251-OR and PEO1-OR) following treatment with lixumistat or FK866, with or without exogenous NAD⁺ supplementation (1mM). Both lixumistat and FK866 exhibited comparable levels of PARylation inhibition, and these effects were effectively restored by the addition of exogenous NAD⁺.

**Figure S8. Migration & invasion assays with parental cells following drug treatment**

Representative images from migration and invasion assays with parental cells (SNU251, PEO1) following treatment with the IC_50_ of olaparib and lixumistat. Quantification of migratory and invasive areas was performed by analyzing four non-overlapping fields at 200× magnification. and data are present as mean ± SD in the bottom panel. Abbreviations: con: control; ola: olaparib; combi: combination; n.s: non-significant. Independent sample t-test: **p* < 0.05, ***p* < 0.01, ****p* < 0.001.

**Figure S9. Migration & invasion assays with parental cells and olaparib-resistant cells following SNAIL knockdown**

A. Immunoblot analysis of epithelial (E-cadherin) and mesenchymal (ZEB1, LAMC2, SNAIL, and vimentin) marker expression in parental cells (SNU251, PEO1) and olaparib-resistant cells (SNU251-OR, and PEO1-OR) following treatment with the IC_50_ of olaparib and lixumistat (based on the parental cells). E-cadherin (SNU251, SNU251-OR) was not detected, consistent with negligible mRNA levels (Ct > 36 in RT-qPCR). Abbreviations: con: control; combi: combination.

B. Representative images from migration and invasion assays with parental cells (SNU251, PEO1) and olaparib-resistant cells (SNU251-OR, PEO1-OR) following SNAIL knockdown. Quantification of migratory and invasive areas was performed by analyzing four non-overlapping fields at 200× magnification, and data are presented as mean ± SD in the bottom panel. Abbreviations: control: control; K/D: knockdown. Independent sample t-test: *p < 0.05, **p < 0.01, ***p < 0.001.

C. Representative images from migration and invasion assays with olaparib-resistant cells (SNU251-OR, PEO1-OR) following exogenous IL-6 supplementation.

D. Immunoblot analysis of SNAIL levels in the olaparib-resistant cells (SNU251-OR, PEO1-OR) following treatment with IL-6 and lixumistat.

E. Representative flow cytometry plots of intracellular IL-6 levels in olaparib-resistant cells (SNU251-OR, PEO1-OR) following SNAIL knockdown using SNAIL siRNA. Cell populations were gated to distinguish between IL-6-negative (red box) and IL-6-positive (black box)

F. Immunoblot analysis of pSTAT3 expression following SNAIL knockdown using SNAIL siRNA in olaparib-resistant cells (SNU251-OR, PEO1-OR).

**Figure S10. Combination of MTT assays with parental cells and olaparib-resistant cells following treatment with control siRNA and olaparib**

A-D. Cell viability (MTT) assay of parental cells [(A) SNU251, (B) PEO1] and olaparib-resistant cells [(C) SNU251-OR, (D) PEO1-OR] following treatment with SNAIL siRNA or olaparib and combination of varying concentrations of olaparib and a fixed concentration of siRNA for 72 h. Data represent three independent experiments with similar outcomes.

**Figure S11. Individual tumor growth inhibition graph with CPDX-013 model**

Individual tumor growth curves were drawn according to treatment groups in the CPDX-013 model.

**Figure S12. Average tumor growth curves showing short and long-term responses to olaparib in the WJO-003 model**

Average tumor growth curves were drawn according to treatment groups in independent study of WJO-003 model. When tumor volumes reached 60-80mm^3^, mice were randomized to control (N=3), olaparib (N=3) groups. Olaparib (50mg/kg) was given via oral gavage once daily for 5 weeks, followed by 50mg/kg twice daily for 5 weeks, and then 75 mg/kg twice daily thereafter. Error bars represent SEM of 2-3 mice per group. Independent sample t-test on day 35 (short-term response) and day 92 (long-term response). Abbreviation: n.s: non-significant. Independent sample t-test: *p < 0.05.

**Figure S13. Individual tumor growth graph with WJO-003 model**

Individual tumor growth curves were drawn according to treatment groups in the WJO-003 model.

**Figure S14. Individual tumor growth inhibition graph with WJO-003-O2 model**

Individual tumor growth curves were drawn according to treatment groups in the WJO-003-O2 model.

**Figure S15. Individual tumor growth inhibition graph with WJO-013 model**

Individual tumor growth curves were drawn according to treatment groups in the WJO-013 model.

**Table S1. List of primary and secondary antibodies which were used in western blot.**

List of Primary and secondary antibodies were used in western blot. Abbreviation: WB, western blot.

| Antibody | Host  species | Dilution | Company (catalog#) | Application | RRID |
| --- | --- | --- | --- | --- | --- |
| Poly/Mono-ADP Ribose | Rabbit | 1:1000 | 83732, Cell signaling | WB | AB_2749858 |
| ZEB1 | Rabbit | 1:1000 | 3396, Cell signaling | WB | AB_1904164 |
| E-cadherin | Rabbit | 1:1000 | 3195, Cell signaling | WB | AB_2291471 |
| SNAIL | Rabbit | 1:1000 | 3879, Cell signaling | WB | AB_2255011 |
| LAMC2 | Mouse | 1:200 | sc-28330, Santa Cruz | WB | AB_2134476 |
| Vimentin | Rabbit | 1:1000 | 5741, Cell signaling | WB | AB_10695459 |
| RB (phosphor-Ser780) | Rabbit | 1:1000 | 8180, Cell signaling | WB | AB_10950972 |
| NRF2 | Rabbit | 1:1000 | 12721, Cell signaling | WB | AB_2715528 |
| γH2AX | Rabbit | 1:1000 | 5438, Cell signaling | WB | AB_1958836 |
| STAT3 | Mouse | 1:1000 | 9139, Cell signaling | WB | AB_331757 |
| STAT3 (phosphor-Tyr705) | Rabbit | 1:1000 | 9145, Cell signaling | WB | AB_2491009 |
| GAPDH | Rabbit | 1:1000 | 2118, Cell signaling | WB | AB_561053 |
| Anti-Rabbit HRP | Goat | 1:500 | GTX213110-01, Gene Tex | WB | AB_10618573 |
| Anti-Mouse HRP | Goat | 1:500 | GTX213111-01, Gene Tex | WB | AB_10618076 |

**Table S2.** **CI values for lixumistat and olaparib in MTT assay**

The CI values at the specified drug concentrations were calculated according to the Chou–Talalay method. CI > 1 indicates antagonism, CI < 1 indicates synergism.

| **SNU251** | | | |
| --- | --- | --- | --- |
| Olaparib (µM) | Lixumistat (µM) | Effect | Combination  Index |
| 30 | 11.5 | 0.604 | 0.702 |
| 15 | 11.5 | 0.526 | 0.703 |
| 7.5 | 11.5 | 0.486 | 0.66 |
| 3.75 | 11.5 | 0.409 | 0.687 |

| **SNU251-OR** | | | |
| --- | --- | --- | --- |
| Olaparib (µM) | Lixumistat (µM) | Effect | Combination  Index (CI) |
| 400 | 13 | 0.688 | 0.553 |
| 200 | 13 | 0.688 | 0.477 |
| 100 | 13 | 0.647 | 0.541 |
| 50 | 13 | 0.613 | 0.592 |

| **PEO1-OR** | | | |
| --- | --- | --- | --- |
| Olaparib (µM) | Lixumistat (µM) | Effect | Combination  Index (CI) |
| 132 | 11.65 | 0.873 | 0.464 |
| 66 | 11.65 | 0.773 | 0.485 |
| 33 | 11.65 | 0.613 | 0.619 |
| 16.5 | 11.65 | 0.471 | 0.726 |

| **PEO1** | | | |
| --- | --- | --- | --- |
| Olaparib (µM) | Lixumistat (µM) | Effect | Combination  Index |
| 10 | 6 | 0.733 | 0.546 |
| 5 | 6 | 0.546 | 0.682 |
| 2.5 | 6 | 0.449 | 0.644 |
| 1.25 | 6 | 0.391 | 0.604 |

**Table S3. CI values for lixumistat and talazoparib in MTT assay**

The CI values at the specified drug concentrations were calculated according to the Chou–Talalay method. CI > 1 indicates antagonism, CI < 1 indicates synergism.

| **SNU251** | | | |
| --- | --- | --- | --- |
| Talazoparib (µM) | Lixumistat (µM) | Effect | Combination  Index (CI) |
| 100 | 11.5 | 0.751 | 0.34 |
| 50 | 11.5 | 0.688 | 0.425 |
| 25 | 11.5 | 0.652 | 0.428 |
| 12.5 | 11.5 | 0.639 | 0.391 |

| **SNU251-OR** | | | |
| --- | --- | --- | --- |
| Talazoparib (µM) | Lixumistat (µM) | Effect | Combination  Index (CI) |
| 200 | 11.5 | 0.654 | 0.532 |
| 100 | 11.5 | 0.588 | 0.669 |
| 50 | 11.5 | 0.573 | 0.515 |
| 25 | 11.5 | 0.562 | 0.416 |

| **PEO1-OR** | | | |
| --- | --- | --- | --- |
| Talazoparib (µM) | Lixumistat (µM) | Effect | Combination  Index (CI) |
| 200 | 11.65 | 0.794 | 0.133 |
| 100 | 11.65 | 0.652 | 0.459 |
| 50 | 11.65 | 0.583 | 0.579 |
| 25 | 11.65 | 0.547 | 0.536 |

| **PEO1** | | | |
| --- | --- | --- | --- |
| Talazoparib (µM) | Lixumistat (µM) | Effect | Combination  Index (CI) |
| 100 | 6 | 0.798 | 0.524 |
| 50 | 6 | 0.775 | 0.473 |
| 25 | 6 | 0.763 | 0.366 |
| 12.5 | 6 | 0.733 | 0.371 |

**Table S4. CI values for lixumistat and olaparib at parental cells’ IC_50_ dose in MTT assay**

The CI values at the specified drug concentrations were calculated according to the Chou–Talalay method. CI > 1 indicates antagonism, CI < 1 indicates synergism.

| **SNU251-OR** | | | |
| --- | --- | --- | --- |
| Olaparib (µM) | Lixumistat (µM) | Effect | Combination  Index (CI) |
| 30 | 26 | 0.749 | 0.34 |
| 30 | 13 | 0.391 | 0.764 |
| 30 | 6.5 | 0.294 | 0.702 |
| 30 | 3.25 | 0.288 | 0.484 |

| **PEO1-OR** | | | |
| --- | --- | --- | --- |
| Olaparib (µM) | Lixumistat (µM) | Effect | Combination  Index (CI) |
| 10 | 23.3 | 0.602 | 0.6 |
| 10 | 11.65 | 0.285 | 0.732 |
| 10 | 5.825 | 0.142 | 0.748 |
| 10 | 2.9125 | 0.114 | 0.568 |

**Table S5. CI values for SNAIL siRNA and olaparib in MTT assay**

The CI values at the specified drug concentrations were calculated according to the Chou–Talalay method. CI > 1 indicates antagonism, CI < 1 indicates synergism.

| **SNU251** | | | |
| --- | --- | --- | --- |
| Olaparib (µM) | SNAIL  siRNA (nM) | Effect | Combination  Index (CI) |
| 30 | 20 | 0.733 | 0.497 |
| 15 | 20 | 0.643 | 0.597 |
| 7.5 | 20 | 0.559 | 0.767 |
| 3.75 | 20 | 0.535 | 0.789 |

| **SNU251-OR** | | | |
| --- | --- | --- | --- |
| Olaparib (µM) | SNAIL  siRNA (nM) | Effect | Combination  Index (CI) |
| 400 | 20 | 0.679 | 0.188 |
| 200 | 20 | 0.445 | 0.914 |
| 100 | 20 | 0.423 | 0.702 |
| 50 | 20 | 0.415 | 0.526 |

| **PEO1-OR** | | | |
| --- | --- | --- | --- |
| Olaparib (µM) | SNAIL  siRNA (nM) | Effect | Combination  Index (CI) |
| 132 | 20 | 0.512 | 0.287 |
| 66 | 20 | 0.391 | 0.501 |
| 33 | 20 | 0.363 | 0.354 |
| 16.5 | 20 | 0.328 | 0.293 |

| **PEO1** | | | |
| --- | --- | --- | --- |
| Olaparib (µM) | SNAIL  siRNA (nM) | Effect | Combination  Index (CI) |
| 10 | 20 | 0.474 | 0.473 |
| 5 | 20 | 0.35 | 0.569 |
| 2.5 | 20 | 0.275 | 0.697 |
| 1.25 | 20 | 0.25 | 0.955 |

**Table S6. CI values for fixed-dose control siRNA and olaparib in MTT assay**

The CI values at the specified drug concentrations were calculated according to the Chou–Talalay method. CI > 1 indicates antagonism, CI < 1 indicates synergism.

| **SNU251** | | | |
| --- | --- | --- | --- |
| Olaparib (µM) | Control  siRNA (nM) | Effect | Combination  Index (CI) |
| 30 | 20 | 0.475 | 1.062 |
| 15 | 20 | 0.341 | 1.097 |
| 7.5 | 20 | 0.216 | 1.283 |
| 3.75 | 20 | 0.209 | 1.068 |

| **SNU251-OR** | | | |
| --- | --- | --- | --- |
| Olaparib (µM) | Control  siRNA (nM) | Effect | Combination  Index (CI) |
| 400 | 20 | 0.609 | 5967.2 |
| 200 | 20 | 0.363 | 431.9 |
| 100 | 20 | 0.287 | 174.8 |
| 50 | 20 | 0.336 | 316.2 |

| **PEO1-OR** | | | |
| --- | --- | --- | --- |
| Olaparib (µM) | Control  siRNA (nM) | Effect | Combination  Index (CI) |
| 132 | 20 | 0.449 | 1.275 |
| 66 | 20 | 0.359 | 1.183 |
| 33 | 20 | 0.29 | 1.091 |
| 16.5 | 20 | 0.274 | 0.925 |

| **PEO1** | | | |
| --- | --- | --- | --- |
| Olaparib (µM) | Control  siRNA (nM) | Effect | Combination  Index (CI) |
| 10 | 20 | 0.044 | 1.251 |
| 5 | 20 | 0.049 | 1.566 |
| 2.5 | 20 | 0.115 | 1.51 |
| 1.25 | 20 | 0.318 | 1.273 |

**Table S7. P-values for tumor volumes at day 22 in the CPDX-013 model, comparing all treatment groups**

Statistical analysis was performed using student’s t-test.

|  | Control | Olaparib | Lixumistat | Combination |
| --- | --- | --- | --- | --- |
| Con | - | 0.058 | 0.240 | 0.009 |
| Ola | 0.058 | - | 0.004 | 0.106 |
| Lixumistat | 0.240 | 0.004 | - | 0 |
| Combination | 0.009 | 0.106 | 0 | - |

**Table S8. P-values for tumor volumes at day 34 in the WJO-003 model, comparing all treatment groups**

Statistical analysis was performed using student’s t-test.

|  | Control | Olaparib | Lixumistat | Combination |
| --- | --- | --- | --- | --- |
| Con | - | 0.425 | 0.723 | 0.126 |
| Ola | 0.425 | - | 0.596 | 0.852 |
| Lixumistat | 0.723 | 0.596 | - | 0.290 |
| Combination | 0.126 | 0.852 | 0.290 | - |

**Table S9. P-values for tumor volumes at day 22 in the WJO-003-O2 model, comparing all treatment groups**

Statistical analysis was performed using student’s t-test.

|  | Control | Olaparib | Lixumistat | Combination |
| --- | --- | --- | --- | --- |
| Con | - | 0.907 | 0.977 | 0.008 |
| Ola | 0.907 | - | 0.929 | 0.029 |
| Lixumistat | 0.977 | 0.929 | - | 0.007 |
| Combination | 0.008 | 0.029 | 0.007 | - |

**Table S10. P-values for tumor volumes at day 21 in the WJO-013 model, comparing all treatment groups**

Statistical analysis was performed using student’s t-test.

|  | Control | Olaparib | Lixumistat | Combination |
| --- | --- | --- | --- | --- |
| Con | - | 0.916 | 0.384 | 0.785 |
| Ola | 0.916 | - | 0.311 | 0.657 |
| Lixumistat | 0.384 | 0.311 | - | 0.419 |
| Combination | 0.785 | 0.657 | 0.419 | - |
