# Supplementary material for: Targeting oxidative phosphorylation with lixumistat overcomes PARP inhibitor resistance in ovarian cancer
Source: Cell Death Dis. 2026 Aug 7;17(1):939. doi: 10.1038/s41419-026-09140-y (PMC13737184; doi:10.1038/s41419-026-09140-y)
Supplement: Supplementary file 2 [file 41419_2026_9140_MOESM2_ESM.docx]

**Original western blots: Figure 1A**

**Original western blots: Figure 1B-C**

**Original western blots: Figure 1E-F**

**Original western blots: Figure 3E**

**Original western blots: Figure 3F**

**Original western blots: Figure 3G**

**Original western blots: Figure 4C**

**Original western blots: Figure 4H**

**Original western blots: Figure 4I**

**Original western blots: Figure 5L-M**

**Original western blots: Supplementary Figure 6B**

**Original western blots: Supplementary Figure 7**

**Original western blots: Supplementary Figure 9A**

**Original western blots: Supplementary Figure 9A**

**Original western blots: Supplementary Figure 9A**

**Original western blots: Supplementary Figure 9A**

**Original western blots: Supplementary Figure 9D**

**Original western blots: Supplementary Figure 9F**
